# Nanoparticles: Taking a Unique Position in Medicine

**DOI:** 10.3390/nano13030574

**Published:** 2023-01-31

**Authors:** Tomy Muringayil Joseph, Debarshi Kar Mahapatra, Amin Esmaeili, Łukasz Piszczyk, Mohamed S. Hasanin, Mashhoor Kattali, Józef Haponiuk, Sabu Thomas

**Affiliations:** 1 Department of Polymer Technology, Faculty of Chemistry, Gdańsk University of Technology, G. Narutowicza, 80-233 Gdańsk, Poland; 2 Department of Pharmaceutical Chemistry, Dadasaheb Balpande College of Pharmacy, Nagpur 440037, India; 3 Department of Chemical Engineering, School of Engineering Technology and Industrial Trades, University of Doha for Science and Technology (UDST), Arab League St, Doha P.O. Box 24449, Qatar; 4 Cellulose and Paper Department, National Research Centre, Cairo 12622, Egypt; 5 Department of Biotechnology, EMEA College of Arts and Science, Kondotty 673638, India; 6 International and Inter-University Centre for Nanoscience and Nanotechnology, Mahatma Gandhi University, Kottayam 686560, India

**Keywords:** biomaterials, medicine, pharmaceuticals, diagnosis, biomaterials, biomedical, nanomaterials, nanoparticles

## Abstract

The human nature of curiosity, wonder, and ingenuity date back to the age of humankind. In parallel with our history of civilization, interest in scientific approaches to unravel mechanisms underlying natural phenomena has been developing. Recent years have witnessed unprecedented growth in research in the area of pharmaceuticals and medicine. The optimism that nanotechnology (NT) applied to medicine and drugs is taking serious steps to bring about significant advances in diagnosing, treating, and preventing disease—a shift from fantasy to reality. The growing interest in the future medical applications of NT leads to the emergence of a new field for nanomaterials (NMs) and biomedicine. In recent years, NMs have emerged as essential game players in modern medicine, with clinical applications ranging from contrast agents in imaging to carriers for drug and gene delivery into tumors. Indeed, there are instances where nanoparticles (NPs) enable analyses and therapies that cannot be performed otherwise. However, NPs also bring unique environmental and societal challenges, particularly concerning toxicity. Thus, clinical applications of NPs should be revisited, and a deep understanding of the effects of NPs from the pathophysiologic basis of a disease may bring more sophisticated diagnostic opportunities and yield more effective therapies and preventive features. Correspondingly, this review highlights the significant contributions of NPs to modern medicine and drug delivery systems. This study also attempted to glimpse the future impact of NT in medicine and pharmaceuticals.

## 1. Introduction

Nanoparticles (NPs) usually refer to particles 1 to 100 nm in size and the catalytic and adsorptive properties are very good for nanoparticles of certain materials. Nanomaterials are also known as “zero-dimensional” NPs. This definition is based on the fact that all of their dimensions are in the nanoscale, as opposed to one-dimensional nanomaterials (NMs) (such as nanowires and nanotubes), which have one dimension larger than the nanoscale, and two-dimensional NMs, which have two dimensions larger than the nanoscale (such as self-assembled monolayer films). They are categorized into numerous classes based on their properties, shapes, and sizes. Fullerenes, metal NPs, ceramic NPs, and polymeric NPs are among the different groups. Because of their large surface area and nanoscale size, NPs have distinct chemical and physical properties. Their optical properties are said to be size-dependent, imparting distinct colors due to absorption in the visible area. Many features that are unique to nanoparticles are directly related to particle size. As a result, efforts have been undertaken to capture some of these qualities by introducing NPs into composite materials. The modern rubber tire is an example of how the special features of nanoparticles have been used in a nanocomposite material, which is often a mixture of a rubber (an elastomer) and an inorganic filler (a reinforcing particle), such as carbon black or silica NPs. Despite manufacturing challenges, the usage of nanoparticles increased significantly in the early twenty-first century, with nanocomposites (NCP) leading the way. NCPs were used in the creation and design of novel materials, serving as building blocks for new dielectric (insulating) and magnetic materials, for example. Other materials show unique optical properties, for example, solar panels used to make ultra-thin films of organic materials. Such batteries are relatively inexpensive but quantum efficient, and also flexible. Synthetic NPs can react with natural nanoscale—well-refined NPs of proteins and nucleic acids can self-assemble into particular structures. Such a structure contains strictly ordered nanoparticles and often exhibits unusual features [1].

On the other side, an NP is known as a quasi-zero-dimensional (0D) nano-object in which all characteristic linear dimensions have the same size and, as accepted worldwide, nanoparticles are spheroidal. The nanoparticles with the apparent discretion of the energy level are often referred to as “quantum dots” or “artificial atoms” and often, they have the structure of ordinary semiconductor materials. Otherwise, some NPs are performed as pseudomonas with complex internal systems, a core and shell, and often outer working groups [2]. Their unique magnetic properties appear to be 2–30 nm in size. Because of their size limit, nanoparticles, like any other particles, begin to vary sharply from the total they cause when they reach a specific size [3]. They are divided into three main classes: three-dimensional (3D) particles obtained by blasting conductors, plasma aggregation, and thin films; two-dimensional (2D) materials—films obtained by molecular layering methods, ionic method layering, etc. Uniform materials—whiskers, these materials achieve molecular layering method, the introduction of materials into cylindrical micropores, etc. [4]. There are also NCP—objects obtained by introducing nanoparticles into any matrix. Currently, only the microlithography method is widely used, which makes it possible to obtain flat island objects up to 50 nm in size on the surface of the matrix; it is used in electronics; the CVD and ALD method is used primarily to create micron films. Other methods are mainly used for scientific purposes. In particular, ionic and molecular layering methods should be considered, as they can be used to create monolayers. In contrast to bulk materials, many of the physical and chemical properties of NPs depend heavily on their size, so in recent years there has been considerable interest in solutions for NP size measurement methods: NP trajectory analysis, dynamic light spectroscopy, dynamic light analysis, and analysis [5].

The numerous applications of NPs and nanomaterials (NMs) in materials ranging from adsorbent and water treatment to biomedical applications include polymers, food packaging, flame retardants, batteries and supercapacitors, nanoceramics, light control, and medicine. Because of their optical, electric, and structural features that cannot be observed in microscopic molecules or macroscopic solids, NMs are becoming increasingly relevant. Assembling NPs into nanostructures allows for the creation of new NMs with novel or enhanced physical and chemical properties and applications. Various researchers are using plant-mediated biological approaches to synthesize nanoparticles of metal, metal oxides, and other materials with varying sizes, shapes, and quantities due to their ease of availability and eco-friendliness. NMs can be synthesized using a variety of approaches, including physical, chemical, biological, and hybrid techniques. These nanoparticles are being studied for a variety of applications, including potent antimicrobial agents, electrochemical sensors and biosensors, medicine and healthcare, agriculture, crop biotechnology, pests, nutrients, and plant hormones, as well as their in vitro anticancer efficiency. Table 1 summarizes the cellular impacts of NPs in diverse cellular systems based on exposure doses, particle concentration, particle size, cell line type used, and the principal outcome of each study.

This study attempts to emphasize the significant contributions of NPs to modern medicine while also discussing the environmental and societal implications of their use. This review is intended to serve as a broad introduction to the role of nanoparticles in medicine rather than as an exhaustive review. Furthermore, this study will concentrate on technologies that have proceeded to the clinical application or in vivo testing. This review will explore instances of medical applications of NPs within the broad areas of medical imaging and drug/gene delivery. Wherever feasible, the reader will be directed to the numerous extensive reviews that are currently accessible in each application area. Finally, the environmental and societal consequences and challenges of using NPs in modern medicine will be examined.

## 2. Generic Features of Nanoparticles in Medicine

### 2.1. Self-Organization of Nanoparticles

One of the essential questions facing nanotechnology (NT) is how to group and organize molecules in a particular way to obtain new materials or equipment finally. For that, the NP regulators emerged to solve the NP self-organization and enhance it as well. Polysaccharides and other biopolymers such as polylactic acid (PLA) were used in this regard [50,51,52]. Instead of focusing on specific molecules, it explores how different molecules associate with one another to form new things. It is encouraging that such systems exist in nature and similar processes occur. Therefore, known biopolymers can organize themselves into separate structures. Particles or NPs in the order of nanometers, as they are called in scientific circles, have a property that dramatically hinders their use. They can form agglomerates, i.e., cling to each other. Manufacturing industries offer NPs, ceramics, and metallurgy; this problem needs to be solved. A possible solution is the use of substances—dispersed ammonium citrate (aqueous solution), imidazoline, and oleic alcohol (insoluble in water). They can be added to a medium containing NPs [53].

### 2.2. Nanoparticles and Healthcare

The general opinion of experts is that researchers have not yet developed the tools needed to 100% assess the risks associated with the role of NPs in healthcare. Such developments are 3–5 years old and, by some estimates, lag far behind even in the actual creation of essential medical NPs. NPs are a completely new product, and the nature of the hazard to human health and the environment is imperative in all circumstances. NPs have a complex set of physical and chemical properties and biological effects (including toxicity) that differ radically from the properties of the same substance in the form of continuous phases or macroscopic expressions [54]. NPs are a modern concept that has gained widespread popularity in various fields of human life. NPs are still being studied today. Therefore, micro consolidates its position in science and thereby contributes to the development of many of its branches, such as pharmacology and medicine. We do not yet know as much as we would like about NPs, but scientists work hard on this issue [55]. NPs are isolated, environmentally pronounced borders and spheroid-shaped objects 1–100 nm in size. NMs with sizes ranging from 2 nm to 30 nm are visible. The NP size limit indicates significant differences from the original part when resizing. The practice has shown that they observe evident changes in sizes below 30 nm. With the help of special equipment, particle size analyzers can measure NPs. This device is in demand in various industries and technological processes and is very simple to operate and accurate in terms of performance. Another attractive property of NPs is their ability to detect human fingerprints and increase the likelihood of detection at crime scenes [41]. Australian and American researchers suggested this method. The use of NPs with “bound” antibodies reveals the fraction of amino acids.

NPs can protect the human body from various diseases and electromagnetic radiation. Specialists in the development of new technologies in the United States have announced that they have found an alternative to antibiotics in the form of polymer NCPs, polymer conjugates, enzymatic modifications and recognition of the structure and properties, functionalized NPs, prodrug polymeric nanoconjugates encapsulating gold NPs, biopolymer-based membranes, biosorbents, and nanotherapeutic agents to combat the disease. Pharmacists argue that it is impractical to develop many antibiotics in our time because they require substantial financial costs. After all, the bacteria are addicted to the drug, gradually converting. Currently, scientists are working on developing NPs, the main task of which is to fight various infections [56,57,58,59,60,61,62,63,64].

Polymeric NPs have been intensively explored as particulate carriers in the pharmaceutical and medical areas because their controlled—and sustained—release capabilities, subcellular size, and biocompatibility with tissue and cells make them promising as drug delivery methods [65,66,67]. Forming drug-loaded particles with sizes ranging from 1 to 1000 nm is the process of nanoencapsulation [68]. Drugs can be absorbed directly from the surface of the sphere or enclosed within the particle [59,63,69,70,71,72,73,74,75,76].

### 2.3. Nanoparticles and Human Health

**Figure 1:**
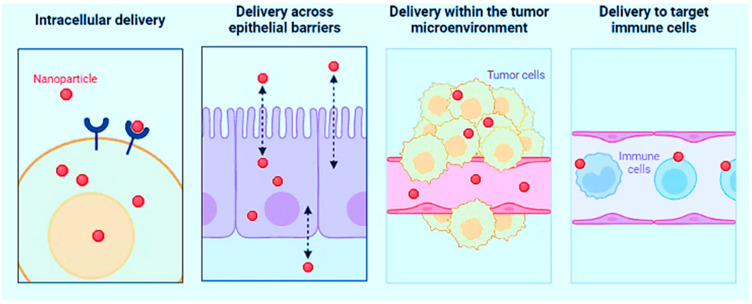
Biological barriers that nanoparticles can help overcome.

NPs can enter the human body in various ways: (i) through the lungs, where they can quickly travel through the bloodstream to crucial organs, including crossing the blood–brain-barrier (BBB), and (ii) through the digestive tract or skin. The material’s biocompatibility is vital. Noble metals such as gold, platinum, and palladium are incredibly biocompatible, silver is moderately biocompatible, and titanium is extensively utilized in implants because it is biocompatible. Silicon crystals are not biocompatible [77]. Although biocompatible, luminous semiconducting QDs frequently include hazardous arsenic or cadmium. Evident Technologies’ indium gallium phosphide (InGaP) QDs is exceptions [78]. NPs with a diameter of fewer than 100 nm accumulate in the alveolar area of the lungs [79]. The same is true for nano-fibers with small diameters. As long as the particles themselves do not disrupt the clearance mechanism, the lungs can clear spheroidal NPs with a retention half-life of 70 days. On the other hand, fibers can remain in the lungs for years, increasing the chances of lung cancer. The uptake of NPs in the intestine is more understood than in the lungs or on the skin, and it is vital for designing NPs for medicine delivery and food stabilization [80]. The nature of the surface, size of the nanomaterial, and entry location are critical factors in determining whether NPs can penetrate the human body beyond the lungs. If the NPs are harmful after entering, the nature of the surface becomes more important [81]. Surface radicals and reactive oxygen species, for example, are assumed to play a crucial role in developing fibrosis and lung cancer by this substance [82]. Other sunscreen ingredients, such as ZnO and titanium dioxide (TiO_2_) NPs, may function similarly and hurt the underlying skin. Figure 1 illustrates the biological barriers that NPs can help overcome.

### 2.4. Biomedical Nanoparticles

Most NMs clinical trials underway or on the market are injected and provide passive medication targeting. When creating NMs, the stability of nanocarriers in biological media is critical [83]. Their fast clearance and aggregation must be avoided, and their pharmacokinetic characteristics should be investigated beforehand [84]. Attaching ligands [RGD, antigen, and folate] that specifically recognize and bind to the target site is currently the most general strategy to boost its affinity toward a target. As a result of their extravasation through permeable vasculature, this active targeting, which relies on particular interactions, can lead to the nanoplatforms accumulating preferentially in tumor regions, ischemic tissue, or inflammatory areas, an effect known as increased permeation and retention (EPR). For the practical application of regenerative therapies, multifunctional NPs have been used to construct biomaterials and nanoplatforms that can entrap and deliver medications and biomolecules (such as DNA and growth and differentiation factors, among other bioactive agents) to cells and tissues. GNPs coupled with a DNA–polymer combination, for example, have been employed as nanoscaffolds for the reverse transfection of DNA into hMSCs [85]. They have also been used to reinforce or boost the bioactivity of polymeric matrices in 3D tissue engineering scaffolds. Nanocarriers intended for delivery can overcome biological barriers such as cell membranes and the blood–brain barrier and carry high drug concentrations of medicines to be delivered [86]. Therapeutic medications must be transported from the nanocarrier once the target location has been reached to become bioavailable and promote the regeneration process. Diffusion, particle erosion, particle degradation, and polymer swelling are passive mechanisms that can influence the release. The nanoplatform must be sturdy enough to allow controlled cargo release only when triggered. Intriguingly, in vivo signals such as pH, ion concentration, redox potential, the presence of particular enzymes, and temperature can be used to initiate the delivery. Exogenous triggering with NMs that respond to light, magnetic fields, or ultrasounds can be beneficial because it allows you to manage the timing and degree of release, reducing drug release at non-target locations [87].

## 3. Applications of Nanoparticles in Medicine

### 3.1. Silver Nanoparticles

**Figure 2:**
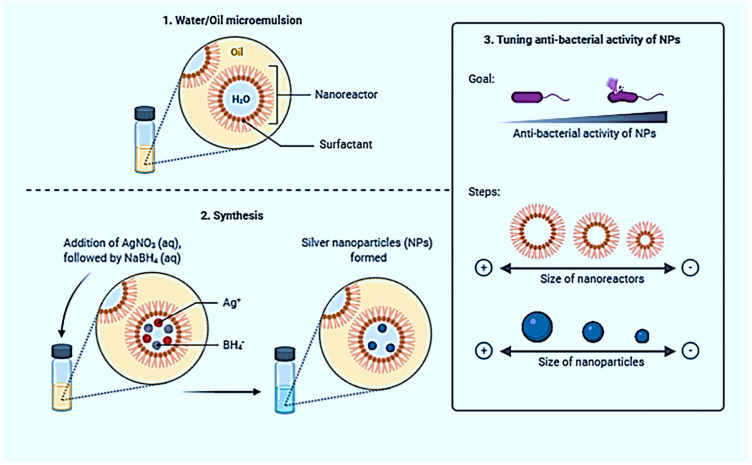
Silver nanoparticles with anti-bacterial activity are synthesized via microemulsions. Water-in-oil microemulsions can serve as nanoreactors for synthesizing nanoparticles such as silver. Interestingly, the size of the nanoreactors (aqueous core) can be easily varied via adjustment of the water-to-surfactant molar ratio that can generate nanoparticles with controllable size and composition. Such control could have a pronounced effect on the antibacterial activity of silver nanoparticles.

First, it has unusual bactericidal and antiviral activity. Humans have long known the antimicrobial properties inherent in silver ions. Water flows through a silver filter that does not contain many pathogenic bacteria found in normal water. Therefore, it can be stored for years without deteriorating. In addition, such water has a particular concentration of silver ions, which can neutralize harmful bacteria and microorganisms, which explains the effect on human health. Nano Silver powder has a high specific surface area and efficiency high-efficiency autocatalytic activity. It is absorbed on the surface of bacterial cells and interferes with the normal metabolism and reproduction of bacteria. It is widely used in medical and everyday textiles and health care products; Nano Silver powder can replace precious metal powder to make an electronic paste with better performance [88,89,90]. This technology can promote further optimization of microelectronics processes. Figure 2 shows the silver NPs with anti-bacterial activity synthesized via microemulsions.

Silver NPs are thousands of times more effective at fighting bacteria and viruses than silver ions [91,92,93,94]. Experiments have shown that the density of NPs kills all known microorganisms (including the AIDS virus) without destroying them. Furthermore, unlike antibiotics, which have compromised activity against harmful viruses and infected cells, the action of the NPs is highly selective: they act only on the viruses and do not damage the cell! The fact that the shell of a microorganism contains specific proteins stops it from supplying oxygen to the bacteria when the NPs break down. Unfortunate organisms can no longer “fuel” glucose to oxidize and die, leaving it without a source of energy. Viruses that have no envelope acquire NPs when they encounter them. However, human and animal cells have more “high-tech” walls, and NPs are not afraid of them. Currently, research is underway into the possibilities of using silver NPs in pharmaceuticals [95]. Yet now they are finding a lot of applications.

**Figure 3:**
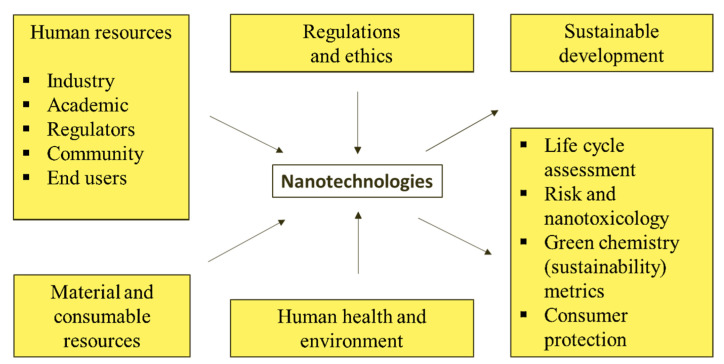
Key issues in dealing with the potential impact of nanotechnology on human health.

For example, the “Helios” company “Witch Doctor” toothpaste is made of silver NPs, effectively protecting against infections. In addition, small concentrations of NPs are added to some creams from a series of “elite” cosmetics to prevent discoloration during use. Silver NP additives are an antiallergenic preservative in lotions, shampoos, and cosmetics. When used, it has an anti-inflammatory and healing effect. Modified fabrics using silver NPs are self-disinfecting by default. Not even a pathogenic bacterium or virus can “coexist” with them. NPs are not washable when washed; their practical life is >6 months, indicating unlimited possibilities for use—fabrics in medicine and everyday life. Materials containing silver NPs are indispensable for medical gowns, bed linen, children’s clothing, and anti-fungal shoes. After application to many hard surfaces (glass, wood, paper, ceramics, metal oxides, etc.), NPs can retain their bactericidal properties for a long time. This allows for the creation of long-lasting disinfectant aerosols that are highly effective for home use. Unlike bleach and other chemical disinfectants, NP-based aerosols are non-toxic and do not harm human or animal health. Issues relating to minimizing the impact of NT on human health and the environment are summarized below [96,97,98,99]. Key issues in dealing with the potential impact of NT on human health are classified in Figure 3.

People are always looking for ways to fight infections transmitted by influenza, tuberculosis, meningitis, and viral hepatitis [100,101,102]. However, our apartments and offices, especially in crowded places (hospitals, public institutions, schools, kindergartens, barracks, prisons, etc.), are full of pathogenic microorganisms that infected people expel. Traditional defense methods do not always solve this problem, so nanochemists have come up with a brilliant solution: add silver NPs to paints and varnishes covering the walls of institutions. When this changed, most pathogenic microorganisms could not “live” on walls and ceilings painted with such colors. As in the case of conventional silver ions, the NPs added to the carbon filters for water practically do not wash away, and this is what the term implies.

In short, tiny, opaque, eco-friendly silver NPs can be used wherever cleanliness and hygiene are required. At the same time, as the leading scientists in the field assure, the funds and materials created on their basis will not be much more expensive than traditional analogs, and with the development of NT, they will be available to all. Samsung is already adding silver NPs to cell phones, washing machines, air conditioners, and other home appliances. In addition to disinfecting properties, silver NPs also have high electrical conductivity, making it possible to create various conductive adhesives. Conductor glue can be used in microelectronics to connect small electronic components. Other application of nanosilver powder includes (1) *Anti-bacterial substances:* With its high specific surface area and efficiency high-efficiency autocatalytic activity of nanosilver dust, it is absorbed into the surface of bacterial cells and inhibits the normal metabolism and reproduction of bacteria. It is widely used in medical and everyday textiles and health products; (2) *Driving paste:* Nano Silver powder replaces precious metal powder to make an electronic paste with better performance. This technology can promote further optimization of microelectronics processes; and (3) *Catalyst:* Improve the speed and efficiency of chemical reactions, such as ethylene oxidation and alcohol oxidation to aldehydes.

### 3.2. Nano Zinc Oxide

ZnO NPs have many unique properties (including bactericidal properties, cytocompatibility, and high safety profile toward human skin) [103], which have the potential to absorb a wide range of electromagnetic radiation, including ultraviolet, infrared, microwave, and radio frequencies [104,105,106,107]. For example, such particles can protect from ultraviolet rays and give new functions to glass, plastics, paints, and synthetic fibers. These particles can also prepare sunscreens, ointments, and other preparations, as they are safe for humans and do not irritate the skin. The ability of ZnO NPs to emit electromagnetic waves makes them invisible in the infrared range by absorbing the heat emitted by the human body using clothing. From radio to ultraviolet radiation, it is possible to create hidden blinds at wide frequencies. Such clothing cannot be changed during military or counterterrorism operations, as it allows you to approach enemies without seeing night vision equipment. High-purity ZnO NPs are designed for use in electronics, catalysts, medical products, and personal care products [108,109,110]. Table 2 shows some bio-imaging applications of ZnO NPs.

ZnO exists in the Earth’s crust as the mineral zincite, however, the majority of it used commercially is synthesized. ZnO is non-toxic and skin-compatible, making it an acceptable addition for fabrics and surfaces in touch with flesh. In comparison to bulk ZnO, the increasing extent of nanoscale ZnO has the potential to improve fabric operation potency. As a vital semiconductor with significant scientific and technological interest, ZnO has a large exciton-binding energy (60 meV) and on-the-spot wide gap (3.37 eV), making it the most popular multifunctional metal and chemical compound with an enormous list of attractive features [105]. UV light-emitting devices, ethanol gas sensors, photocatalysts, pharmaceutical, and cosmetic industries all make extensive use of ZnO-NPs.

### 3.3. Nano Silica

**Figure 4:**
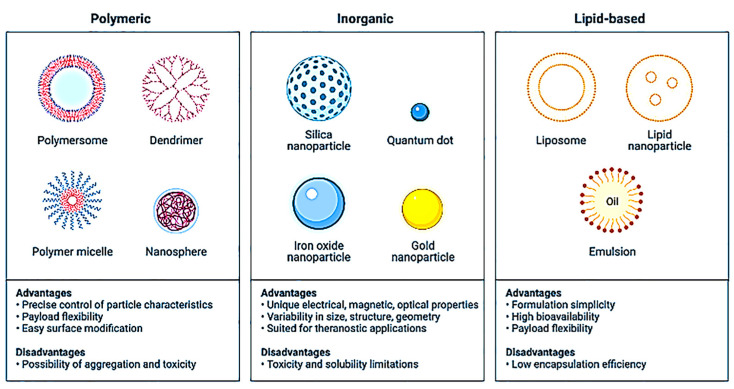
Various classes of nanoparticles.

Silicon dioxide (SiO_2_) NPs have a fantastic property: if applied to any material, they attach to its molecules, allowing surfaces to expel dirt and water [120,121,122]. Self-cleaning nano-coatings protect glass, tiles, wood, and stone based on these particles. Dirt cannot stick or penetrate the protected surface; water flows easily from it and removes any dirt. Dendritic fibrous nano-silica (DFNS) has also been reported to have biological applications such as cell therapy, gene therapy, immunological therapy, drug delivery, imaging, photothermal therapy, bioanalysis, biocatalysis, and tissue engineering [123]. Figure 4 shows various classes of NPs.

## 4. Nanotechnology in Pharmaceuticals and Medicine

The targeted design industry for new drugs or drug design is directly related to NT because reactive substances such as drugs and targets represent molecular substances [124,125]. The basic concepts used in drug design are target and drug. Target is a macromolecular biological structure that is associated with a specific function, the violation of which leads to disease and requires a particular influence. The most common targets are receptors and enzymes. A drug is a chemical compound that specifically interacts with the target and modifies the cellular response that creates the target in one way or another. If a receptor acts as a target, the drug will most likely be its ligand, i.e., a compound that interacts exclusively with its active site. For example, F1-adenosine triphosphates (F1-adipose) are a group of enzymes that synthesize energy in all organisms, including photosynthesis in plant cells. The diameter of the enzyme molecule is 10–12 nm.

Supermolecules are associated with two or more chemical particles bonded by intermolecular non-covalent bonds from geometric and chemical correspondence fragments. The rearrangement of molecules leads to a variety of combinations. Such systems include supermolecular chemistry (the term Nobel laureate J., for example, the use of a porous structure that plays the role of a “host” in other cases, this role is usually played by an organic ligand), a “guest” in nanoscale for selective transport and drug release. Supermolecular structures are undoubtedly a suitable object for further study of nanocrystals. A system of proteins up to UM) that provides a ligand–biojet complex (subscript–receptor or host–guest type), a supermolecular structure (supermolecular component) of all known components (system) [126,127,128,129].

By continuing these considerations, the therapeutic nanoscale effect of a drug targeted at a bio target can only be achieved if a supermolecular nanosystem “ligand-biotarget” is formed. The development of targeted drugs falls under the definition of NT given above, as their mode of action is based on the targeted interaction with the disease-causing bio target [130,131]. This interaction with the nanoscale is understood through the non-covalent (coordination with hydrogen) between the drug (ligament) and the protein (target) studied during development, which determines the selectivity, efficacy, and low toxicity of the targeted drugs. Previous generation drugs, i.e., improves consumer behavior. Moreover, during its existence, the “ligand-biotarget” system, in all its characteristics, is a machine, and the result of its action will be a modification of the disease (complete or partial treatment). Therefore, the efficacy of nanomedicine (NM) depends on the strength and duration of binding to the complex components under discussion, which, for a consistent purpose, depends solely on the properties of the innovatively targeted ligand drug. Then, by formalizing the concepts, it can be argued that NT in pharmaceuticals is a set of methods and techniques for studying, designing, producing, and using, the main stages of which need to be considered.

- Biological screening, i.e., the search for active molecules (1–10 nm) that interact with a bio target (protein or a system of proteins up to 100 nm in size).
- Study the mechanism of action (search for a bio target and identify the mechanism of the reaction of an active molecule).
- Computer design of compounds that can be activated by estimating the energy of interaction between candidate molecules several nanometers apart and a bio target (protein), that is, calculating the possible structures and positions of the molecules corresponding to the minimum energy of such reaction at approximately 24 h of dynamic simulation of roughly 200 h.
- Component nanoscale targeted control and modification of components’ shape, size, reactivity, and integration (“ligand-biotarget”, approximately 1–100 nm), resulting in enhanced or additional functionality of products and consumer features (efficacy, bioavailability, and side effects of innovative drugs that reduce toxicity).
- Nano-sized finished dosage forms (liposomal forms, biodegradable polymers, NPs for targeted transport, etc.).
- The use of innovative targeted drugs that have a nanoscale effect on a bio target, leading to a therapeutic effect.

### 4.1. Medicine and Pharmaceuticals in Nanoscience

**Figure 5:**
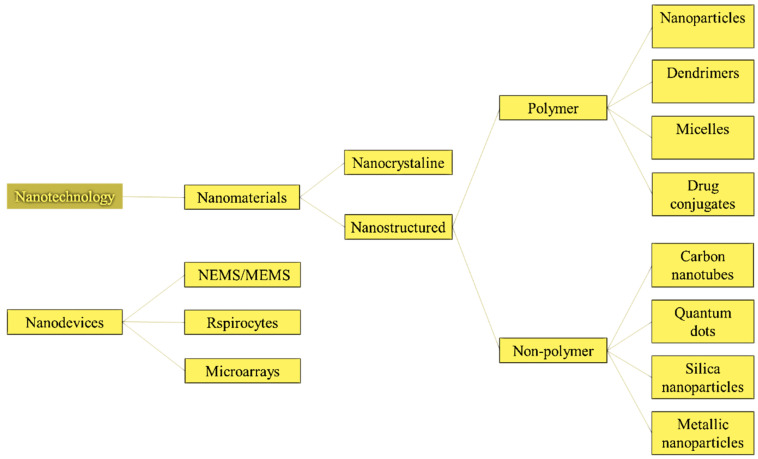
Schematic diagram of various types of pharmaceutical nanosystems.

NT is an interdisciplinary field of fundamental and practical scientific technology that targets theoretical justification, techniques, and methods for the study, design, production, and use of nanostructures, tools, and systems and the shape, size interplay, and integrated control of their components [132,133,134,135]. Nanoscale components (approximately 1–100 nm) obtain materials with new chemical, physical and biological properties. NT can help create an object by manipulating the individual atoms of matter. The replacement of other technologies will defeat old age and disease and provide human beings with incredible material wealth. NT solves the following vital tasks in medicine, pharmaceuticals, and related fields through the creation of solids and surfaces with modified molecular structures. It supplies metals, inorganic and organic compounds, nanotubes, biologically compatible polymers (plastics), and other materials that mimic living tissue and serve as vehicles for drug delivery or implants. Figure 5 denotes the schematic diagram of various types of pharmaceutical nanosystems.

### 4.2. Nanostructured Materials Uptake by the Human Body

In the form of future medication, NT in the human body is a game-changing notion that will drastically alter the treatment of malignant diseases that cause anatomical alterations in the human body. NT is a new subject that is predicted to have a significant role in various medical fields. Because of nanomaterials’ high potential for toxicity, regulatory organizations are cautious about approving new nanomaterials for human usage. The many nanocarriers used to transport and distribute active chemicals to target tissues should be treated as enhancers, as they may have detrimental side effects or dissolve or agglomerate within the body. The growing interest in new nanostructured materials with potential medical uses has not been matched by adequate knowledge of these materials. Many types of nanocarriers (inorganic and organic) are employed to transport therapeutic drugs to cancer sites, and biomedical NT has arisen as a solution for numerous therapeutic difficulties [136,137,138]. One promising drug carrier is the PLA-PEG (polylactic acid-poly(ethylene glycol)) multiblock copolymer. Several anticancer medications have been encapsulated and linked with PLA-PEG copolymer NPs, including chemotherapeutic agents, aptamers, nucleic acids, and peptides. Because of their stealth, NPs can elude the immune system. They can also be dispersed for longer after injection, increasing their longevity. The application of PEG increases the deliquescent of several chemotherapeutic hydrophobic medicines, resulting in enhanced solubility [139]. Various anticancer drugs have been encapsulated and linked with PLA-PEG copolymer NPs, including chemopharmaceuticals, aptamers, nucleic acids, and peptides [140,141,142].

Various researchers are investigating the controlled distribution of biosurfactants via PLA-PEG copolymeric NPs. Folic acid is used as a ligand to target cancer cells’ overexpressed folic acid receptors, allowing for more precise dosing. The cytotoxicity of nanoformulations (NFs) against the MDA-MB-231 breast cancer cell line is studied. By causing apoptosis, biosurfactants induce cancer cells to die. Polymeric NPs’ release kinetics is certainly determined (in vitro). Based on this research, biosurfactants produced from *Candida parapsilosis* infused in polymeric NPs could be examined as a potential therapeutic technique. The effect of biosurfactant-loaded NPs on the proliferation of the MDA-MB-231 breast cancer cell line was investigated. Fickian diffusion is a mechanism for releasing biosurfactants from the polymer matrix utilized in kinetic models. The folate ligand was also combined with the PLA-PEG copolymer to achieve active model targeting in cancer cells. Most internalization and cytotoxicity against a breast cancer cell line (MDA-MB-231) are seen here compared to non-targeting formulations. Furthermore, this formulation was discovered to induce apoptosis in a breast cancer cell line, resulting in the death of malignant cells. PLA-PEG polymeric NPs can be employed as a container for the regulated release of a new biosurfactant produced from *C. parapsilosis* to inhibit breast cancer cells [143,144].

There were several methodologies recommended at the beginning for the precise delivery of non-carcinogenic drugs to the cancerous targeted site, including the direct application of an anticancer drug to the tumors or infected area, systemic delivery targeted to the tumor drug delivery to the blood vessels, anti-neoplastic drug formulations and carriers, and transmembrane drug delivery, as well as biological therapies [145,146]. Nanotherapeutics for cancer are still evolving, and they are being used to solve a variety of problems with standard medicine. Anti-neoplastic medications are encapsulated in nanocarriers and transported to a specific tumor before being released, resulting in effective drug moiety absorption within the tumor cell. The introduction of the porous metal-organic compound (MOC) greatly impacted numerous scientists’ studies. Metal ions or clusters and organic ligands are used to self-assemble porous crystalline materials into frameworks. Because of its advantages, including high specific surface areas, configurable pore size, and customizable internal surface properties, MOC is used in various potential applications. Adsorption or separation, catalysis, and drug administration are only a few applications. MOCs are exploited as carriers for stimuli-specific medication delivery because of these intrinsic features.

### 4.3. Development of Nanomedicine (NM)

**Figure 6:**
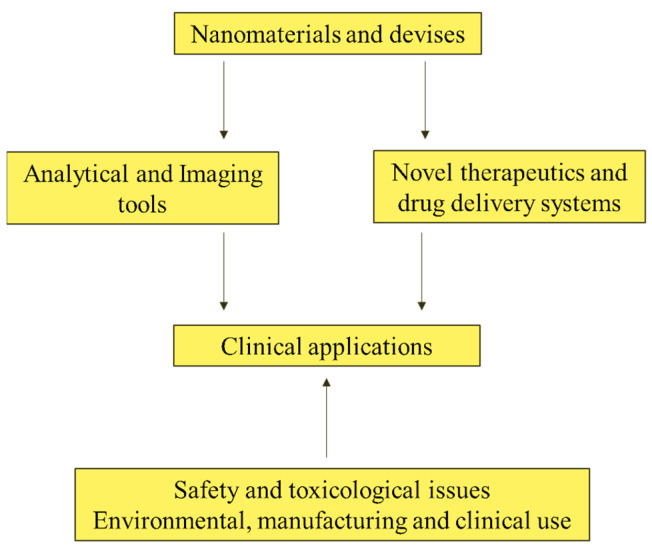
Dimensions in nanomedicine.

The potential for using NT in medicine is ultimately the need to change the cell structure at the molecular level using nanorobots or other NT [147,148]. In recent years, NM has been developing extraordinarily and has attracted public attention for its natural benefits and social contribution. The term (which also reflects perspective) means NT in diagnosing, monitoring, and treating diseases. The development of NM has been associated with revolutionary advances in genomics and proteomics, which have allowed scientists to come closer to understanding the molecular basis of diseases. NM is developing at the nanometric level by creating materials with new properties that combine genetics and proteomics data with capabilities. There are five main areas for applying NT in medicine: supply of active drugs, new methods, and therapies at the nanometer level, in vivo diagnostics, in vitro diagnostics, and medical implants. Figure 6 shows the dimensions of NM.

### 4.4. Nanobiotechnology and Drug Discovery for Personalized Medicine

**Figure 7:**
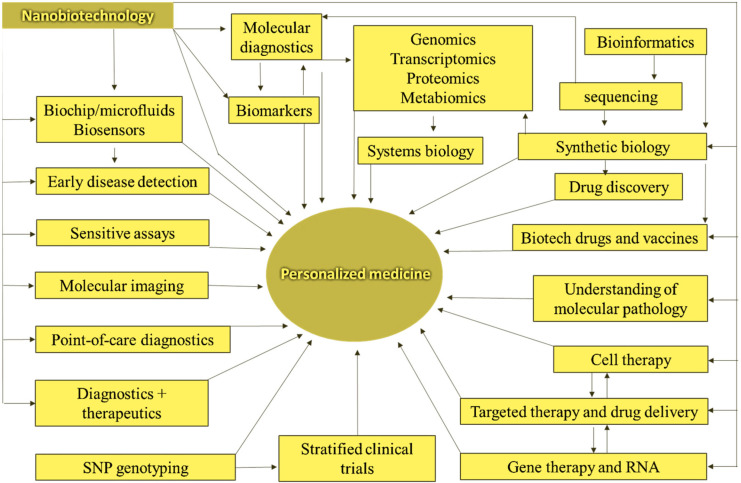
Nanobiotechnology and drug discovery for personalized medicine.

Personalized medicine refers to specialized treatments and medications most suited to an individual [149]. Individualized or individual-based therapy is another name for it. Personalized medicine is founded on considering a patient’s genotype to determine treatment options (along with other considerations). Nanobiotechnologies (NB) are already being employed in molecular diagnostics, an essential component of customized medicine. Personalized medicines could be found and designed for specific groups of patients using pharmacoproteomics. Current efforts utilizing pharmacogenomics and pharmacogenetics include matching existing treatments to the proper people for optimal efficacy and safety. In addition to aiding the integration of diagnostics and therapies, NB promises to facilitate the identification of tailored medicines. Figure 7 shows the classifications of NB and drug discovery for personalized medicine. The figure details how NB will facilitate the integration of diagnosis and therapy, which is an essential part of personalized medicine. The most critical applications are in the management of cancer.

### 4.5. Physical, Chemical, and Microbiological Parameters Affecting Nanomedicine

Physical, chemical, and microbiological parameters such as physical appearance and morphology, particle size and size distribution, surface charge and surface chemistry, drug content and pH value, drug loading and release kinetics, and in vitro degradation and dissolution are commonly analyzed during stability testing for all types of NMs (targeted/non-targeted) [150,151]. In some circumstances, drug leakage studies have documented instability testing (i.e., liposomes). Most thermal stability methods were tested between 4 °C and 37 °C. The duration of storage was not closely followed in these investigations. However, according to ICH guidelines, it should be up to 12 months (long-term storage). Additional stability parameters for NMs combined with biotechnological products (e.g., cancer-targeting ligand-conjugated NMs) are suggested in the ICH guideline Q5C. The potency of the biotechnological product, dissociation of active moieties from NMs, purity, and molecular characterization are the additional factors applicable, according to ICH guideline Q5C. The stability protocol should include these criteria to guarantee optimum safety and quality. Despite the benefits of employing NPs for applications where properties such as targeting, release, and encapsulation are advantageous and the creation of “smart” therapies or personalized medicine, the number of FDA-approved NP-based treatments, medications, and devices is very minimal. Toxicity, product repeatability, generic bioequivalence, biodistribution, proper human clinical trials, and appropriate and detailed characterization methodologies are essential aspects of the NM field [152,153,154,155]. The difficulties encountered during development and function evaluations point to NM’s limitations, divided into four categories: biology, manufacturing, safety, and cost. Because several review publications highlight the issues connected with the usage of NPs, this document will only go over a few of the limits and methods to overcome them.

#### Prodrugs

The prodrug technique, which dates back over 50 years, is covalently attaching a drug to a polymer scaffold or macromolecule to help pharmaceuticals bypass physiological barriers [156,157]. To release the actual medication, prodrugs must undergo chemical or enzymatic transformations in the body. A moiety is a nontoxic detachable protective group attached to an active drug molecule to improve the medication’s transport or effectiveness; it usually is promptly removed from the body when the prodrug unit is activated. The prodrug platform creates more potent treatments by boosting aqueous solubility, decreasing toxicity effects, enhancing site-specificity, increasing cell absorption, and diminishing the “burst release” impact of many drug delivery strategies. 

### 4.6. MRI

A typical MRI strategy is to physically encapsulate contrast chemicals into polymeric NPs [158]. T1 and superparamagnetic iron oxide NP [SPION (T2)] MR agents can be encapsulated and delivered using polymeric NPs. Responsive conditions might release the encapsulated chemicals into a target location. To confirm the use of NPs as a targeted agent for early detection of acute pancreatitis, enzyme-activated fatty acid-coated gadolinium diethylenetriamine penta-acetic acid (GADTPA-FA) NPs were produced. The release of Gd-DTPA fatty acid NPs increased the imaging signal in in vitro and in vivo investigations [159]. SPIONs have been encapsulated using self-assembled amphiphilic polymers in several studies. A polymeric micelle system could be a viable choice for SPION medication delivery and targeted MRI. However, as previously stated, responsive polymers are not suitable candidates for clinical applications due to their complexities. Another extensively utilized approach for making polymer NPs is the covalent coupling of contrast agents. Using covalently cross-linked polymers allow experiment with different combinations to improve relaxivity, circulation, and contrast agent degradation. The stability, biocompatibility, and imaging effects of several covalently linked Gd-based polymeric NPs have been investigated for MRI applications. For example, Liu and colleagues proposed employing multifunctional antivascular endothelial growth factor (anti-VEGF)-linked PLA-PEGpoly(L-lysine) (PLL)-Gd NPs to target liver tumor cells to detect hepatocellular carcinoma early. Gd-loaded polymeric NPs treated with anti-VEGF considerably increased the targeted delivery of Gd to VEGF-positive liver tumor cells, according to both in vitro and in vivo data. Shalviri et al. developed a multifunctional terpolymer system capable of simultaneously delivering contrast agents and anticancer medicines to a specific spot in another investigation [160,161,162].

### 4.7. The Position of Drugs and Bioactive Molecules in the Nanometer World

In 1959, the famous American theoretical physicist R.J. Feynman said, “There is a wonderfully complex world of small forms, and one day (for example, in 2000) people will be surprised that no one took research seriously until the 1960s.” Medicine and pharmaceuticals are among the most important practical applications of nanotechnologists in this world because the world described above is the world of these disciplines. These dimensions characterize the main biological structures—cells, components (organs), and molecules. For the first time in medicine, the concept of microscopic devices (which should include NPs) was introduced by R. J. Feynman in his famous speech, expressing the idea that “there is plenty of space.” In recent years, Feynman’s proposals have come closer to reality. However, they are still far from the micro boat he suggested, capable of penetrating the heart through the circulatory system, performing a valve operation there, and performing similar imaginative procedures [160,163,164,165].

The stated perspectives illustrate that today’s specific work of NT in medicine can be divided into several groups: nano-relief surfaces, nanostructured materials including nano-porous skins; NPs (including fullerenes and dendrimers); micro-nanocapsules (NCs); NT sensors and analyzers; medical applications of scanning probe microscopes;nanotools and nano manipulators; micro–nano devices of different levels of autonomy. The application of the “nano” (Greek, part billion) material indicates that their dimensions are within 10^−9^ m, which corresponds to the dimensions of the biological organization from atomic to subcellular. Thus, practically any supermolecular complex, i.e., molecules (in modern terms, “host”) of ionic or covalently formed molecules (“guest”) of “small and large” molecules. However, according to the tradition already established in the biological and medical literature, NPs mean perfectly specific (and, above all, artificial) molecular structures [166,167,168].

In his review published a few days ago, researchers from the United States (US) and France were forced to modify the term “NP”. They believe that these particles need to be more precisely organized for further research and practical application in various fields. Although similar suggestions have been heard, one cannot agree with this view. Experts say the essential idea is not to assign new materials to NPs “blindly according to their size”, but based on whether this size leads to the appearance of new properties of such materials. Although NMs have already found widespread application in many countries, even in cosmetics and sunscreens, there are no clear laws governing the safe use of NPs in the same countries. In contrast, without a clear definition of “NP,” it is not appropriate to expect such forms of laws. Although there is an opinion that a nano-object should be considered an object, its dimensions should be less than 100 nm.

The review’s authors argue that NPs cannot be classified as “they are all rowing in the same brush” However, not all “small” particles are certainly NPs. The question arises as to what criteria should be used when processing NPs. The review examines the physical and chemical properties underlying the proposed new classification. For example, the size of a nanosystem affects its crystal structure, which determines the specificity of NPs’ interactions with the environment. For example, the properties of NPs as large as 10–30 nm are significantly different from those of more giant formations.

### 4.8. Development of Nanocontainer Technologies for Vector Drug Distribution

Synthesis of new chemical compounds by creating molecules without chemical reactions. Over the next 10–20 years, this will lead to fundamentally new drugs that will be “designed” by synthetics, pharmacists, and doctors based on a particular disease, even a specific patient. Develop self-replicating (self-replicating) systems based on biosimilars—bacteria, viruses, and protozoa [169,170].

### 4.9. Creation of Accurate Medical Nano Manipulators and Diagnostic Tools

Nanotechnologists are developing methods to construct objects with specific properties from these parts, considering an individual atom as a component. Many companies already know how to combine atoms and molecules into one structure. In the long run, any molecule will fit together like a child’s construction set because any chemically stable system that an appropriate formula can describe can be built [171,172].

## 5. Nanoparticles in Drug Delivery

**Figure 8:**
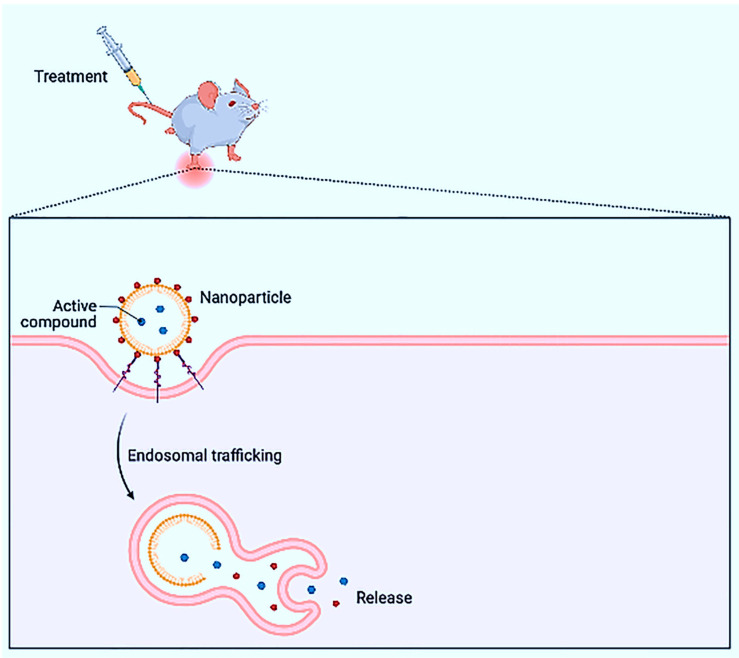
Nanoparticle drug release mechanism.

Depending on the physicochemical features of the medication, it can be physically trapped within the hydrophobic cores of micelles, similarly confined in the aqueous space, or intercalated into the lipid bilayer of liposomes. The drug of interest is dissolved, entrapped, attached, or encapsulated throughout or within a polymeric matrix in nanospheres. Antigen-loaded nanospheres have the potential to be used in vaccine delivery. NPs can be used to deliver drugs across the BBB. Anti-cancer medications, DNA, and vaccinations can be given via natural polymers such as chitosan. As gene carriers, chitosan–DNA NPs can be employed. Because of their bioavailability in deep lung tissue and ease of distribution, NP aerosols are appealing for drug delivery. SWCNTs can carry drugs, antigens, and genes, and functionalized SWCNTs with DNA attached can boost uptake and expression more than free DNA [173,174,175,176]. Figure 8 shows the drug release mechanism of NP.

### 5.1. Delivery Systems for Biologically Active Materials

Transdermal (through the skin) is one of the simplest and most effective means of transporting drug molecules into the human body [177,178]. There are no theoretical restrictions on delivering this type of known biologically active compound, regardless of its molecular weight (size) or physicochemical properties. However, for the nano transporters described below, the transdermal method is considered one of the possible ways to deliver the NPs (the figure shows NPs used to deliver therapeutic molecules: 1: liposome and adenovirus; 2: polymer NP; 3: dendrimer (DD); 4: carbon nanotube. Liposomes of various components and multi-components are formed in lipid solution. Liposomes larger than 20–50 nm used to deliver drugs to a biological target may be desirable for practical purposes. In addition, nature itself has developed many nanocarriers, for example, viruses. Of course, treated adenoviruses can be used effectively for transdermal vaccination. In addition to liposomes, synthetic biogenic NPs capable of targeted delivery include lipid nanotubes, NPs of lipid origin, nanoemulsions, NPs from certain cyclops, chitosans, and nucleic acids distributes bactericidal drugs as nanobiomachines [179,180,181,182,183].

It has already been proven that bacteria can be used to transport drugs to infected tissues. Experts carried the MC-1 bacteria into the rat’s blood. These bacteria can scurry due to the rotation of the flagella, but in addition, they contain magnetic NPs, which sensitize the magnetic field and force them to travel through energy lines. They are capable of generating such force records as magnetic resonance devices. Researchers believe that before attempting to create synthetic nanomachines that can travel through the human body, we should focus on creations that already exist in nature.

Nanospheres and nanocapsules (NC) belong to the family of polymer NPs. If the nanospheres are solid matrices, the polymer shell in the NCs forms a chamber filled with liquid on the surface of the polymer that distributes the active substance. As a result, the active substance is released into the body through various mechanisms—the release from nanospheres is exertional, and from NCs—it occurs at a constant rate for a long time. Polymer NPs can be obtained from natural or synthetic polymers, such as polysaccharides, polylactic and polyglycolic acids, polylactide, polyacrylates, acrylic polymers, polyethylene glycol, and their analogs: biodegradation and functional compatibility [184].

Of particular interest is the DD, which represents a new type of polymer with a “branching” structure, not the usual linear. The first sample was returned in the 1950s, and primary synthesis methods were developed in the 1980s. The term “DD” appeared long before “NT” and was initially unrelated. Recently, however, DD has become increasingly popular in the context of its nanotechnological and nanomedical applications. DDs are a unique class of polymers during chemical synthesis because their size and shape can be specified very accurately, which is very important for NPs. DDs can be obtained from monomers by continuous convergent and divergent polymerization (including peptide synthesis methods), thus setting the branching pattern. The most common monomers used in the synthesis are polydopamine and the amino acid lysine. “Target” molecules bind to DDs by creating complexes on their surface or inserting them deep into individual chains. In addition, the desired functional groups can be arranged stereospecifically on the surface of the DDs, which is most effective against viruses and cells. An example of creating an active ingredient based on a DD is Vivigel, a gel that can protect against HIV infection [185,186,187].

Fluorescence is the most widespread carbon NP formed only by carbon atoms and can be obtained using nanotubes and various chemical or physicochemical methods [188,189]. For example, fullerenes can be obtained by thermal spraying of carbon-containing soils on an industrial scale, in an inert gas atmosphere, at low pressure, and in the presence of a catalyst. According to experts, fullerene may be the basis for delivery systems and a new class of drugs. The main feature is their frame shape: the molecules appear closed and hollow “inside the shell”. The most famous carbon framework structure is the C60 fullerene, which made a completely unexpected discovery in 1985, leading to a significant leap in research in the field (the Nobel Prize in Chemistry in 1996 was awarded to those who discovered fullerenes). After developing a method of obtaining fullerenes on a macro level, many others found lighter or heavier fullerenes: C20 to C70, C82, C96, and above. Fullerenes are used to develop drug-delivery vehicles to treat HIV-infected and cancer patients [190,191,192].

In 1991, quite unexpectedly (theorists did not predict their existence), elongated, cylindrical carbon forms were discovered, which obtained their names from nanotubes. They came in various shapes: large and small, single, multi-layered, straight, and spiral. They display a whole range of unique powers, and the most unexpected electrical, magnetic, and optical properties. Nanotubes can be used as microscopic vessels to transport many chemically and biologically active substances: proteins, toxins, fuel components, and molten metals. Nanotubes have an essential relationship with lipid structures; using peptides and DNA oligonucleotides, they can form stable complexes and contain these molecules. The combination of these properties determines their use in the form of effective delivery systems for vaccines and genetic material.

Inorganic NPs, silicon oxide compounds, and various metals (gold, silver, and platinum) are elementary classes of nanocarriers. Such an NP often has a silicon core and an outer shell of metal atoms [193]. Metals make it possible to create carriers with many unique features. Therefore, their action (in particular, the release of a therapeutic agent) can be modulated by thermal exposure (infrared radiation) and changes in the magnetic field. In the case of various solid-phase composites, for example, metal NPs on the surface of a porous support, new features appear due to their reaction.

The most common platform technologies are for obtaining microencapsulation, matrix, multi-layer, shell tablets, and capsules [194]. In Russia, for example, platform technologies have been developed that now holds patents for the creation of nanoscale complexes of active materials using biocompatible, biodegradable synthetic, and natural polymers. A NF can increase the activity of a drug by 2–4 times, with more pronounced therapeutic benefits appearing. In many cases, preliminary studies of drugs known as new nano-packages (e.g., Taxol^®^ or Neurofen with prolonged action) are already underway. Restricted drug release platform technologies are relevant to the targeted delivery of highly toxic anti-cancer drugs. Traditional gynecological drugs are distributed evenly throughout the body: they enter the center of the disease and the healthy organs. Targeted delivery of a drug with a biodegradable polymer can solve the problem, then the drug will not be released instantly, but the polymer will shrink. However, more sophisticated drug delivery methods target at NPs, genetic material, and DNA or RNA [195,196]. Pieces as small as 200 nm or smaller can escape the bloodstream only in areas of inflammation, where the capillary pores become enlarged.

**Figure 9:**
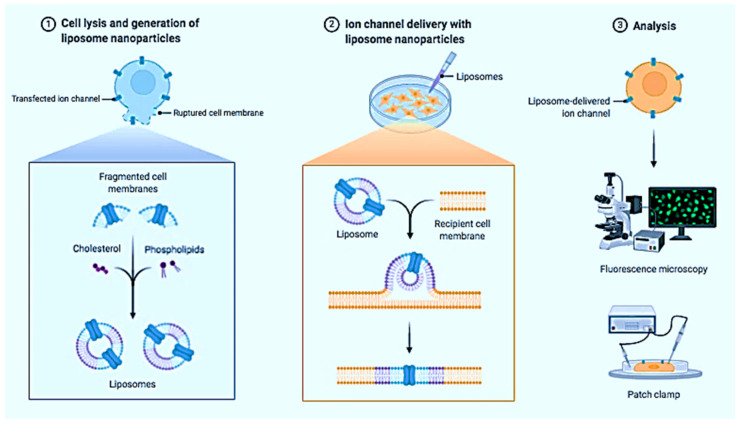
Ion channel malfunctions lead to physiological disorders such as cystic fibrosis. Current therapies are merely symptomatic. To solve this, the bilayer of liposomes is employed to integrate and deliver intact functional channels. Ion channels were collected in the form of membrane fragments to preserve their structure. Phospholipids chose to give liposomes a fusogenic property to place ion channels at their action site. This therapeutic principle was assayed microscopically and electrophysiologically.

During their journey through the bloodstream, NPs proliferate by plasma proteins in the blood and absorb the immune defenses—macrophages [197]. To increase the stay time of the NPs in the body, polymer chains are attached to them. Another option is to adhere to an NP, an antibiotic that destroys the tumor cells, which know the path to the antibody target. For example, scientists are developing a liposomal anti-cancer drug in which thermosensitive liposomes are wrapped in a polymer and equipped with antibodies that determine the “delivery address” [198,199]. Figure 9 shows the liposome NPs for delivery of ion channels.

Many immunizations have become routine procedures against all kinds of diseases, but this technology has not changed in practice in the last century. Soon, syringes containing the antigen solution will be replaced by nano transporters (up to 500 nm in size) that will be able to deliver the antigen through the skin. The use of small NPs (only 40 nm) has been shown to allow the distribution of antigens directly through the hair follicles. At the same time, active drug delivery systems today are associated with risks, i.e., side effects. It is not surprising that the pharmaceutical giant Novartis and Siba’s concerned that some other large companies have linked their further developments in this direction only with biologically destructive nanocarriers.

### 5.2. Nanobiotechnology for a Combination of Drug Design and Drug Delivery

Many previously identified medications could not be employed in patients due to a lack of an appropriate drug delivery system. Drug distribution can also benefit from NT. In 2000, the FDA approved a solid dose formulation of the immunosuppressant sirolimus made with Elan Drug Delivery’s NanoCrystal technology (King of Prussia, PA, USA). Abraxane^TM^ (Abraxis^TM^ Oncology) is an injectable suspension comprising albumin-bound paclitaxel to treat breast cancer after failure of combination chemotherapy for metastatic disease or relapse within six months of adjuvant chemotherapy. It is based on NP technology, which combines biocompatible proteins with pharmaceuticals to produce an NP form of the drug (size 100–200 nm) that overcomes paclitaxel’s insolubility issues. The current trend is to consider medication delivery concerns earlier in drug development and design. NT’s potential applications to aid drug distribution can be regarded during drug development. The medicinal drug and the carrier NP can be created simultaneously. For example, Calando Pharmaceuticals uses NP delivery to create short interfering RNAs (siRNAs) as anticancer medicines. Sequence selection and synthesis of efficient siRNA molecules that attach to (and self-assemble with) the siRNA to produce uniform colloidal-sized particles 50 nm in diameter are used in proprietary technology. Larger particles cannot escape the bloodstream and penetrate the tumor since they are delivered by intravenous injection. If the medication particles are less than 10 nm in size, they are swiftly eliminated by the kidneys [200,201,202,203,204].

### 5.3. Nanotherapy

Nanoscale molecules can also be used as active substances. One of the new enhancements is the breakdown of active medicinal substances into nanometers in size—about half of the fresh active ingredients currently being developed are poorly soluble, meaning they do not have adequate bioavailability [205]. An active drug contains crystals of NPs produced in the form of suspensions (nanosuspensions), which can be given intravenously, and for oral administration, granules and tablets can be created [206,207]. It does not require a polymer matrix, and in the opinion of some scientists, destruction causes toxins in the cells. The typical size of nanocrystals is 200–600 nm. One of the nanocrystalline drugs introduced into clinical practice in 2000 was Rapamon (White-Ayers Laboratories), an immunosuppressive drug used after transplantation. Thermotherapy with NPs seems to have high expectations. It is known that when radiation near the IR reaches the nanotubes, they begin to vibrate and heat the material around them. The effectiveness of such therapy turned out to be very high: in 80% of mice receiving a solution of multi-layer nanotubes, the kidney cancer tumors disappeared after some time. Almost all the rats in this group survived until the end of the study, which lasted for about nine months—clinical trials of thermotherapy for brain tumors and prostate cancer. The researchers found that contact of nanotubes with bone tissue in mice accelerated bone regeneration and reduced the risk of inflammation during treatment. Similarly, nanogold particles (AuNPs) kill microorganisms and identify and destroy cancer cells [208,209,210]. NPs can also be used to stimulate spontaneous reproduction [211,212]. The main focus here is the artificial activation and control of adult stem cells. Several advances include amphiphilic proteins that help cells grow to repair damaged spinal cord nerves; coating of brain tumor areas with magnetic NPs and enzyme-sensitive particles; NP probes for intracellular drug delivery and gene expression, and quantum dots that detect and measure biomarkers of human breast cancer.

Nanoantibodies (NAs) are the smallest (2 × 4 nm in size) molecules identifying the currently known protein antigen. Are fragments of specific single-domain antibodies (variable domains)—they contain a dimer of a hemoglobin-rich immunoglobulin chain and function ideally without a light chain. After synthesis, the NAs are already active, and no post-translational modifications are required. This allows the bacteria to reproduce immediately in yeast cells, making the way these proteins are made more efficient. It is easy to perform all kinds of genetic engineering manipulations with NAs, for example, to create more efficient synthesis structures, including two or more NAs and other protein domains or functional groups. Such antibodies do not exist in the human body and are not compatible with them. Thus, the strategies of past abnormal, pathological cells and microorganisms can be overcome to adapt to the human immune system and find a weak link in their immune system [213,214,215,216,217]. Biologically active additives (BAA), developed with NT, aim to enhance the body’s capabilities substantially: from improving the absorption of active food components to improving mental performance and the ability to focus, the modern market is unique [218]. However, consumer rights societies impose strict government control over products’ actual safety and effectiveness that hit store shelves. Table 3 illustrates the advantages of nanobodies relevant to developing therapeutics.

## 6. Nanoparticles in Pharmaceutical Application Perspectives

Many readers will remember that sunblock was an opaque milky-white ointment a few years ago, the color of which was explained by the presence of micronized zinc oxide particles, which are harmful to the skin due to the ultraviolet part of the sun’s radiation. Straightforward creams are now being made more comfortable and attractive to users. The commercial success of new cosmetic preparations is that they contain identical zinc oxide particles but are broken down into nanometric sizes. These particles still allow most sunlight to pass through but retain their ability to absorb harmful ultraviolet wavelengths. Later, NPs of another well-known white dye (titanium dioxide) began to be used for the same purposes, i.e., the simple replacement of micron particles with nanometric made it possible to create a new and successful commercial product in the cosmetics industry. Changing the properties of titanium dioxide particles allowed them to find another essential technical application called the desensitization of solar cells’ working material. Such batteries primarily determine the efficiency of light conversion by the ability of particles of matter to absorb solar radiation. Titanium dioxide NPs have been found to absorb light thousands of times due to their large area [219,220,221]. These NPs are now widely used in industry, as evidenced by their organization in industrial production in Australia (2001).

Another important commercial market for NPs is related to semiconductor technology [222,223,224]. In the production of chips (microcircuits), there is a process called chemical mechanical planarization (CMP), which, when the required components are applied at several points on the surface of the processed wafer, “applies” to this surface with almost atomic accuracy. Processing a large (300 mm) crystal surface with incredible accuracy is a complex technical task that existing methods cannot solve. In the new form, a suspension of NPs is applied to the surface of the device, which is then used in a combined process of chemical removal and mechanical friction, resulting in the surface being “polished” with atomic precision. This process is very effective when using NPs of many common semiconductor materials (aluminum, silicon, cerium oxides), and as a result, the market volume of products obtained by the CMP method increased from USD 250 million in 1996 to over USD 1 billion in 2000. At the same time, the production of the initial components for the CMP process (suspensions of NPs, polishing machines) naturally became an independent sector of the material market, with a volume of about USD 800 million in 2005. Given the semiconductor industry’s continued trend towards miniaturization and improved matching accuracy, it is sure that the market for products and services related to the CMP process will continue to expand [225,226].

The technologies mentioned above are well known and have already been implemented, but it should be noted that many other technologies are currently undergoing a process of commercialization and technological reform based on the use of NPs. For example, professors Paul Alivisatos (University of California, Berkeley) and MungiBewandi (University of Massachusetts) have proposed new processes for the production of semiconductor NPs from materials such as cadmium selenide (CDC) and cadmium telluride (TES).Parts of this material coated with a layer of zinc sulfide gain the ability to absorb light in the ultraviolet wavelength range and then emit light in the visible spectrum, which is related to the quantum trapping effects, depending on the size of the NPs used for the wavelength of the radiation. Such sources are far superior to known emitters (based on fluorescent chemical dyes) regarding radiation stability and brightness, but they are treasured because NPs can chemically bind to proteins, oligonucleotides, or smaller molecules. NPs give these compounds new functional properties, thus opening up molecules in biological, medical, and biotechnology as fluorescent “tags.” Furthermore, studies have shown that the radiation wavelength of silicon nanocrystals (less than 4 nm in diameter) in the visible range also depends on the crystal size. The emitters created on this basis have become more efficient than fluorescent and other sources currently used in solid-state technology, allowing many possibilities to be applied.

As the size of crystallites decreases to nanometers, their physical and chemical properties (especially catalytic activity) change significantly, a notable example of which is the nature of gold [227,228]. Under normal bulk conditions, gold is known to be a chemically completely inert element. However, at low concentrations (approximately 0.2–0.9%), in the non-metallic form (in the formation of nanoclusters), the particles of cerium dioxide deposited on the surface of gold become highly active catalysts of the known reaction of water-gas conversion, in which carbon monoxide and water are converted, to carbon dioxide and hydrogen. This reaction is vital in the functioning of fuel cells using hydrocarbon fuels, which are then converted into hydrogen and carbon-containing products in such cells. The long-held dream of fuel cell designers and manufacturers is to increase the yield of hydrogen, that is, to reduce the amount of unreacted carbon monoxide, which is the “catalyst toxin” of the electrochemical reaction within the cell. As mentioned, and in minimal quantities, the use of NPs with gold is beneficial from an economic point of view because the content of the noble metal in the catalysts previously used reached 10% [229,230].

Significant changes in the magnetic properties as it goes into the nanoscale are of great interest to researchers, even helping to anticipate the possibility of what may be called superparamagnets [231,232,233]. Superparamagnetic NPs act like ordinary magnets in the absence of a magnetic field and at temperatures above the Curie point, i.e., their magnetic moments are random. However, when an external field is applied, they quickly “line up” with the area, creating a solid general magnetic moment. This system can be used for various purposes, including magnetic resonance imaging (MRI). This method has been known theoretically for a long time, but given the intensity of the images obtained, only a minimal number of natural substances (e.g., deoxyhemoglobin) are produced by the body. The method’s efficiency and image contrast can be significantly enhanced using superparamagnetic iron oxide NPs called SPION (Superparamagnetic Iron Oxide, SPION). Such particles, which are based on magnetite (Fe_3_O_4_), magnetite (gamma Fe_2_O_3_), or combinations thereof, must be coated with a layer of material, increasing the collision system’s stability, and ensuring that the collision system’s stability that biological compatibility with the body. The advantage of the magnetic resonance method is that it allows you to obtain clear images of tissues containing large amounts of fluid (e.g., affected organs or cancerous tumors) [234,235]. Already, such NPs are being manufactured commercially by many organizations. It is understood that the surface of SPION particles can be further chemically modified to enable them to interact with contrast agents, specific tissues, or cell types. This approach is auspicious and has already led to the emergence of areas where various types of biomedical research are actively developing.

### 6.1. Nanoparticles in Medical Imaging

Traditional biological imaging of cells and tissues employing fluorescence microscopy and current magnetic resonance imaging (MRI) of various body locations can benefit significantly from NPs. NPs utilized in these two processes differ in chemical composition. Table 2 summarizes the multiple applications of NPs in imaging. Magnetic resonance, tissue repair, detoxification of biological fluids, drug delivery, imaging contrast enhancement, and hypothermia treatment are all in vivo applications for superparamagnetic iron oxide NPs. Semiconducting QDs have several advantages over organic fluorophores for following cells in vivo at high resolutions. Table 4 explains the summary of current NP technologies in medicine.

### 6.2. Optical Imaging

Organic dyes are loaded into the sample for conventional imaging of cells and tissue sections. Biomolecules linked to dyes such as fluorescein isocyanate (FITC) and rhodamine selectively binds to cells or cell components via ligand/receptor interactions. Inadequate fluorescence intensity and photobleaching are two common issues with this form of imaging. Photobleaching is the slow loss of fluorescence intensity over time caused by irreversible changes in the dye molecules’ chemical structure, rendering them non-fluorescent [236,237]. Quantum dots (QDs) are semiconductor NPs made up of inorganic compounds. Under ultraviolet (UV) lighting, these NPs emit bright fluorescent light, and the wavelength (color) of the fluorescent light released is sensitive to particle size [238,239,240]. These materials have a size dependence that is unique to them. The presence of a “band gap” gives inorganic semiconductor molecules their characteristics. The band gap is the energy difference between the valence band (or energy level) where electrons are typically found and the conduction band, where they can be “encouraged” by a supply of energy of a specific wavelength (excitation), usually in the form of a photon. A “hole” is created when an electron passes from the valence band to the conduction band (a term given to an energy level lacking an electron and not a physical feature). When the excitation stops, electrons return to the valence band, where they release their extra energy. This energy is discharged as light in the case of QDs. Larger QDs have more electron–hole pairs and can collect and release more energy. Because power and wavelength are inversely related (E = hc/λ), the wavelength of produced light drops as QD size grows. Compared to standard organic dyes, QDs can emit light that is substantially more powerful and resistant to photobleaching. This is a significant benefit in 3-D tissue imaging, where a key concern is photobleaching during the acquisition of successive sections in the z-direction.

QDs are insoluble in aqueous solutions since they are inorganic compounds. Therefore, coating QDs with a thin layer of a water-soluble substance is essential for employing them in biological and medicinal applications [241,242]. This is usually followed by a coating with a sense that preferentially binds to a particular cell or cell component. Each QD contains many spots on its surface where soluble bioactive chemicals can be attached. Furthermore, each QD can be connected to several molecules, giving it many functions. Michael and colleagues (2005) highlighted numerous surface modification tactics such as targeting and prolonged retention in circulation in a review of the application of QDs for live-cell and in vivo imaging [243]. The use of oligomeric phosphine-coated QDs to map lymph nodes in mice and pigs was recently disclosed by Kim and colleagues (2004). These QDs are constructed of CdTe capped with CdSe, a combination that can emit light when excited in the near-infrared region. The capacity to map lymph nodes up to 1 cm below the skin surface without the requirement for surgical incisions is significant. The authors extrapolated that the quantities employed were below recognized harmful thresholds because the toxicity of the injected QDs was not evaluated in this investigation [244].

By covering the QD surface with appropriate chemicals, QDs can be targeted to specific organs within the body. By covering the QD surface with a peptide sequence, CGFECVRQCPERC, known to bind to endothelial cells in lung blood arteries, Akerman and colleagues (2002) demonstrated that ZnS-capped CdSe QDs may be guided to the lungs of mice [245]. QDs were directed to blood or lymphatic routes within tumors in animals using the same manner. The QDs were ingested by the targeted cells via endocytosis in both cases but not by cells in the surrounding tissue. Gao and colleagues (2004) encased semiconductor QDs in an amphiphilic copolymer and added targeting ligands to the polymer surface [246]. The QDs were manufactured of CdSe with a ZnS cap and a coordinating ligand to prevent aggregation in the solution (tri-n-octyl phosphine oxide [TOPO]). The copolymer consisted of three segments: butyl acrylate, ethyl acrylate, and methacrylic acid; the first two are more hydrophobic than the latter. The hydrophobic segments of the copolymer are attracted to the TOPO in solution, resulting in the configuration, which has the hydrophilic segment’s carboxylic acid groups protruding out. These acid groups can be utilized to attach molecules such as poly (ethylene glycol) (PEG) or affinity ligands to them. The composite particles had a diameter of 20–30 nm. Tethering an antibody against a prostate-specific membrane antigen to the particle surface enabled tumor targeting (PSMA). Following intravenous injection, these particles selectively bind to tumors in mice. There was no buildup in the brain, kidney, or lungs.

### 6.3. Magnetic Resonance Imaging

The magnetic resonance imaging (MRI) method performs noninvasive 3-D scans of the body [247]. This method is frequently employed in modern medicine, especially in diagnosing and treating most brain, spine, and musculoskeletal illnesses. MRI analyzes hydrogen atoms naturally present in tissue using magnetic resonance spectroscopy (as water and cell membrane proteins, for example). A transverse radiofrequency (RF) pulse ignites the magnetic dipoles within hydrogen nuclei in a sample placed in a strong static magnetic field. The spinning nuclei are aligned with the static field before the RF pulse. The RF pulse gives these nuclei more energy, causing them to spin at a different frequency and in the opposite (transverse) direction. The hydrogen nuclei revert to a condition of equilibrium in alignment with the static magnetic field after the RF pulse. T1 and T2 are two parameters commonly used to describe the relaxation process. T1 is the time required to align nuclear spins with the static field, while T2 denotes the characteristic time during which the nuclei’s transverse magnetization vanishes. Different T1 and T2 relaxation periods can distinguish hydrogen nuclei and various tissue types. MRI scans need the acquisition of many images dependent on spatial location and T1 or T2 weighting. In a T1-weighted picture, a sample with a low T1 appears bright [248].

The inherent differences in relaxation times between regions of interest (such as usual vs. scar tissue) are minor in many therapeutic applications, necessitating the addition of contrast agents. Contrast agents are paramagnetic compounds that can change the relaxation times of specific tissues or fluids in the body. Gadolinium compounds have been successfully used as contrast agents for numerous years, having the capacity to resolve places such as the kidney and brain. Gadolinium-based contrast agents shorten T1. Superparamagnetic iron oxide (SPIO) NPs have recently emerged as potent T2-weighting contrast agents, complementing gadolinium-based agents. T2 weighting is critical for liver, lymph node, and bone marrow imaging. Superparamagnetic NPs (such as iron oxide) have longer relaxation durations than gadolinium-based treatments.

Using a mouse xenograft model, Huh and colleagues (2005) demonstrated how SPIO NPs can be utilized to detect cancer in vivo [249]. The NPs were linked to Herceptin, a cancer-targeting antibody in this study. Before conjugation with Herceptin, SPIO NPs were generated by the thermal breakdown of iron acetylacetonate and made water-soluble by binding with 2,3-dimercaptosuccinic acid. When given intravenously to mice, a fast change in the T2-weighted MRI signal from the tumor in the animals’ thigh was seen. The same iron oxide NPs were coupled to a nonspecific antibody in a control experiment, proving antibody binding specificity. The MRI signal was unaffected by these control NPs.

SPIO NPs can also be utilized to visualize features that are not visible with standard MRI. Harisinghani and colleagues (2003) used SPIO NPs to detect tiny lymph node metastases in human prostate cancer patients [250]. In this example, the NPs were coated with dextran to ensure their retention in the bloodstream and progressive absorption into lymph nodes, where macrophages ingest them. SPIO NPs, enable the imaging of metastases that standard MRI can only hazily detect. The relevance of this research is that individuals with localized cancer can receive early surgery instead of being limited to radiation therapy, which is the standard treatment for advanced-stage cancer patients.

### 6.4. Cancer

NPs have significantly impacted cancer treatment, as indicated by the clinical trials of several NP-based medicines and delivery methods [251,252,253]. Recent reviews have provided examples of various liposome and polymer-based drugs and therapeutic agents. Paclitaxel is a well-known anti-cancer drug used to treat ovarian, cutaneous, esophageal, and lung cancers. This medication inhibits cancer cell activity by stabilizing microtubules, eventually leading to apoptosis. This water-insoluble medicine is most commonly administered as an ethanol solution (Taxol^®^), combined with a polyoxyethylated castor oil (Cremophor EL) solvent. The side effects of Cremophor, including hypersensitivity reactions, necessitated the use of steroids and antihistamines as pre-medications, which was a fundamental flaw in this method. Abraxane, a distinct type of paclitaxel, was licensed for clinical usage in early 2005. In a high-pressure emulsification method, paclitaxel is loaded into albumin NPs, a natural polymer. This soluble version of paclitaxel has been found to eliminate the harmful effects of Cremophor but also to provide some additional advantages. Compared to Taxol^®^, the albumin carrier improves drug delivery from the bloodstream to the tumor site and allows for larger drug doses.

However, paclitaxel NP loading did not address multidrug resistance, an issue that emerges when cancer cells adjust to stimuli by producing efflux transporters or other proteins on the surface [254]. Koziara and colleagues (2006) aimed to overcome this difficulty by putting paclitaxel into emulsifying wax NPs [255]. Tween 80 is a commercially available wax known as polyoxyethylene 20-sorbitan monooleate. NPs were made by emulsifying a resin, medication, and surfactant mixture that had been heated. The efficacy of these drug-loaded NPs was tested in a mouse xenograft model (HCT-15), where tumor cells exhibit the efflux transporter p-glycoprotein (Pgp). The subsequent step of tumor growth was considered to be owing to a combination of overcoming resistance (via non-specific cytoskeletal disruption) and the antiangiogenic impact of paclitaxel with the support of a control experiment employing Taxol^®^. These examples of multiple paclitaxel versions show how various NP-based drug delivery techniques can be used to adjust and improve a medication’s performance.

**Figure 10:**
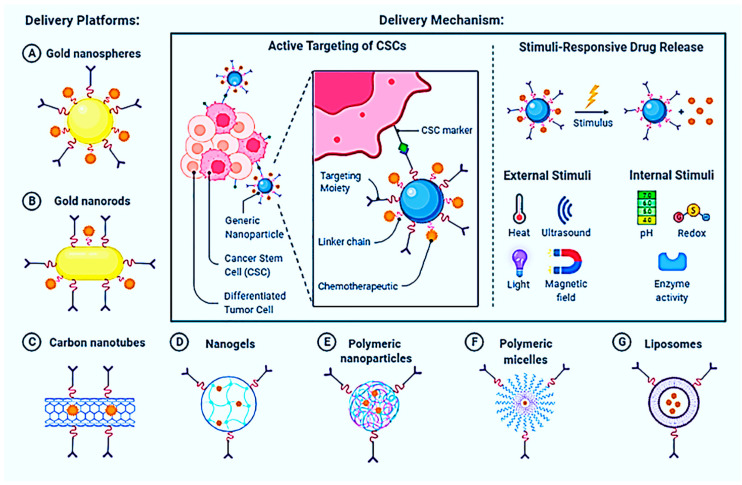
Nanoparticle-mediated targeted drug delivery to cancer cells. Surface markers specific to cancer stem cells (CSCs) serve as potential therapeutic targets for the elimination of CSCs. Active targeting can be achieved by conjugating targeting moieties to drug-carrying nanoparticles. These moieties can specifically bind to the markers expressed on CSCs. Following binding, drug release can be triggered by external and internal stimuli. Multiple nanoparticle-based drug delivery platforms have been developed, utilizing these strategies, as shown in Figure (**A**–**G**).

The interaction of chemotherapeutic and antiangiogenic drugs is significant in tumor therapy [256]. According to Sengupta and colleagues (2005), disruption of tumor blood arteries can affect chemotherapeutic agent delivery and promote increased expression of drug resistance-related variables [257]. These researchers created a two-layer NP drug delivery system with a poly (lactic-co-glycolic) acid (PLGA) core conjugated with doxorubicin and a liposome consisting of phospholipids conjugated with PEG and combretastatin. Doxorubicin is the chemotherapeutic agent, and combretastatin is the antiangiogenic agent [258]. The size of these multilayered particles was 80–120 nm. The main idea was to get the particles to the tumor and then degrade the PLGA core to release the drug slowly. When given intravenously to mice with tumors induced by carcinoma or melanoma cells, the particles were readily taken up by tumors which are consistent with the increased residence time caused by PEG conjugation and the known “leakiness” of tumor vessels (also known as the enhanced permeability and retention, or EPR, effect; tumor vessels have 400–600 nm pores). NPs inhibited tumor growth dramatically, allowing the mice to survive longer. Figure 10 explains the NP-mediated targeted drug delivery to cancer cells.

### 6.5. Neurodegenerative Diseases

In developing effective treatments for neurodegenerative illnesses, drug transport to the central nervous system remains difficult. Overcoming the natural tendency of the BBB to hinder drug transport is an essential aspect of this difficulty. This barrier protects the brain from external drugs and blood-borne illnesses, but it cannot recognize many therapeutic molecules. As a result, substantial doses must be given, increasing the likelihood of adverse side effects. NP-based systems, spanning from polymer particles to liposomes, have been one of the many ways investigated in recent years to overcome this constraint.

**Figure 11:**
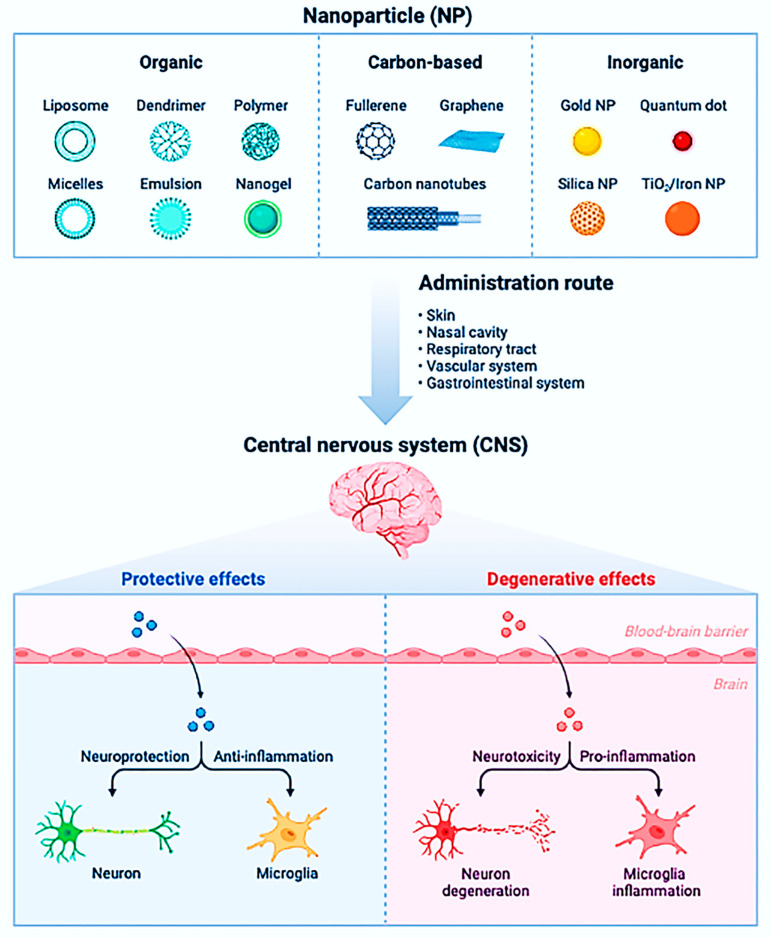
The impact of different types of nanoparticles in the central nervous system. NPs are mainly classified into three groups: organic, carbon-based, and inorganic NPs. In general, these NPs are administrated via the gastrointestinal system, respiratory tract, nasal cavity, skin, etc. They cross the blood–brain barrier (BBB) into the target brain cells including neurons, microglia, and astrocyte to exert protective and degenerative effects.

Drug transport through the BBB has been demonstrated using NPs synthesized from poly(hexadecyl cyanoacrylate) and similar compounds. Kreuter and colleagues (2003) coated poly(butyl cyanoacrylate) (PBCA) NPs with dalargin (an analgesic) and exhibited BBB penetration in rats [259]. In a mouse model of Alzheimer’s disease, Siegemund and colleagues (2006) demonstrated that PBCA NPs loaded with thioflavins can target fibrillar amyloid [260]. Calvo and colleagues developed an NP system made of a PEG-poly(hexadecyl cyanoacrylate) copolymer (PHDCA) [261]. Because PEG is hydrophilic and PHDCA is hydrophobic, the copolymer molecules arrange into particles with an insoluble PHDCA core and a PEG surface layer in an aqueous environment. Many drug delivery methods use PEG because it is not recognized as a foreign material by macrophages, increasing the half-life of drug carriers in the blood. Indeed, the addition of PEG improves PHDCA’s ability to traverse the BBB. Copolymers of PEG and compounds comparable to PEG, such as polyethyleneglycol, can create polymeric micelles (propylene oxide). Pluronic P-85 polymer is a commercially available example, and P-85 micelles have been used to transport analgesics over the BBB in mice. Figure 11 shows the impact of different types of NPs on the central nervous system.

Drug delivery to the central nervous system using liposome-based devices has also been studied extensively. In these systems, PEG surface coverage is also adequate. Liposomes accumulated to significant levels in the central nervous system in mice with experimental autoimmune encephalomyelitis (an animal model for MS) after intravenous injection. The drug-loaded liposomes caused BBB integrity to be restored and a reduction in inflammation and macrophage infiltration. This medication was more effective than giving free glucocorticosteroids, a standard MS treatment. Gene delivery across the BBB is another application of PEG-conjugated liposomes. This strategy is being used to discover treatments for chronic neurological illnesses that do not respond to small-molecule medications (such as Huntington’s disease, Rett syndrome, and Fragile-X syndrome, to name a few). The liposomes were delivered to specified locations using antibodies, and plasmid-induced gene expression in the brain was found for at least six days after liposome administration. The importance of this method is that it allows researchers to transport genes that endonucleases would ordinarily destroy in vivo by loading them into liposomes with targeting capabilities.

### 6.6. HIV/AIDS

pH-sensitive NPs are produced from a copolymer of methacrylic acid and ethyl acrylate to deliver CGP 70726, an HIV-1 protease inhibitor. Eudragit L100–55 is the brand name for this copolymer. Because of its pH-dependent solubility, the copolymer was chosen. CGP 70726 and other antiviral drugs have been shown to stop HIV-1 from replicating. Poor water solubility is a severe issue when administering medications such as CGP 70726. De Jaeghere and colleagues made NPs by emulsifying a copolymer solution with a CGP 70726 and benzyl alcohol mixture. The NPs were given to dogs orally, and blood samples were analyzed to confirm successful drug release [262]. The HIV-1 Tat protein has been identified as a potential candidate for an HIV-1/AIDS preventive or therapeutic vaccination [263]. A cationic lipid and a surfactant were used to create SLNs. Electrostatic forces subsequently adsorbed DNA and Tat peptide onto the NP surface. Increased gene expression was detected when delivered to the lungs of mice by intratracheal instillation or aerosol application, indicating effective transfection. While there are fewer reports of in vivo NP-based medication delivery in HIV/AIDS than in cancer and neurological illnesses, there is undoubtedly activity in the area, as seen by multiple recent in vitro studies [264,265].

### 6.7. Ocular Diseases

The fundamental rationale for employing NP-based drug delivery methods in ophthalmic applications is the capacity to lengthen medication residence periods by trapping the drug in the ocular mucus layer. This layer, which acts as a macromolecule diffusion barrier, is released by goblet cells in the conjunctiva and protects the cornea’s epithelial layer. The majority of ocular illnesses are treated with eye drops containing medication solutions. Because of the rapid pre-corneal loss induced by mucus flow during blinking, these solutions are usually highly concentrated and require frequent administration. The report showed non-steroidal and anti-inflammatory medicines (flurbiprofen and ibuprofen) to rabbit eyes using commercially available Eudragit^®^ polymers. These medications are commonly used to reduce the inflammatory response after ocular surgery. The Eudragit RS and RL polymers employed in these studies were poly(ethyl acrylate), poly(methyl methacrylate), and poly(methyl methacrylate) copolymers (chloromethyl-aminoethyl-methacrylate). These polymers are a good platform for controlled release since they are insoluble and can swell under physiological conditions. Drug and polymer mixtures were mixed in ethanol and emulsified (with water and a surfactant) to generate drug-embedded NPs with a diameter of 100 nm. The rabbit eyes’ conjunctive sacs were injected with saline solutions of these NPs. Both medicines (flurbiprofen and ibuprofen) were put into NPs, which significantly suppressed inflammatory responses following surgical trauma and were comparable to traditional eye drop controls. This result is significant because the NP system was built with a lower medication concentration than the eye drop control. Second, due to the prolonged residence period of the drug in the polymer matrix, the NP method generated more significant drug levels in the tumor [266,267,268]. Drugs must be administered to the retinal portion of the eye in some illness states, such as cytomegalovirus (CMV) retinitis [269,270]. CMV infection can permanently damage the retina, choroid (the area behind the retina), iris, and surrounding tissue. The use of bovine serum albumin (BSA) NPs to carry ganciclovir, a medicine used to treat CMV infection. The drug was incubated in an aqueous solution with BSA, and droplets were formed by adding ethanol in an emulsification procedure; the resulting NPs had a diameter of about 280 nm. These NPs were resuspended in saline before being injected into the eyes. According to the researchers, the NPs persisted in a thin layer on the retina for up to two weeks after injection, and histological investigation revealed no inflammatory reactions or changes in tissue morphology compared to normal eyes [271,272].

### 6.8. Respiratory Diseases

NP-based medication delivery techniques in treating respiratory illnesses have been restricted. Nonetheless, various examples of medicines are beneficial in treating allergies and hereditary and infectious diseases in the respiratory system. In a mouse model of allergic asthma, a liposome-based NP technology was used to reduce inflammation. P-selectin receptors on active endothelial cells in circulation were inhibited, which reduced interactions between endothelial cells and leukocytes. As a result, peribronchial inflammation is less likely to develop. By adding fucose and sulfate ester groups to the liposome surface, the NPs (average diameter of 73 nm) were designed to imitate the physiological P-selectin super ligand (PSGL-1). LPS and cockroach antigens produced lung inflammation and airway hyperreactivity in mice. The liposomal NPs were found to bind preferentially to selectins on activated endothelial cells. When comparing mice treated with NPs to controls, histological analysis revealed a significant reduction in peribronchial inflammation and airway hyperreactivity. The report showed that chitosan/interferon-pDNA NPs, a polymer–drug conjugate, can diminish allergen-induced airway inflammation. It is well understood that allergic illnesses (such as asthma) decrease interferon-α (IFN-α) production in individuals, making them vulnerable to airway inflammation and hyperresponsiveness. Different NPs have been explored to match this demand, such as lipid, liposome, protein, carbon-based, polymeric, metallic, oxide, and magnetic NPs [273,274,275].

### 6.9. Neurology and Nanoparticles

The prevalence of neurological disorders with unknown causes is rising, including well-researched conditions such as Parkinson’s disease, Alzheimer’s disease, and multiple sclerosis. The blood–brain barrier protects the brain, but because medications are required to cross it to reach the lesions, it also makes it difficult to treat and diagnose certain neurological illnesses. Experimental ENPs (metallic NPs, quantum dots, and carbon nanotubes) can enter the brain through a variety of entry sites, according to animal studies (skin, blood, respiratory pathways). A very small portion of the inhaled or instilled ENPs reach the blood and subsequent secondary organs, including the CNS, at a low translocation rate after being inhaled or instilled into respiratory tract portions. Numerous forms of ENPs can have a range of biological impacts on the nervous system, according to experimental in vivo and in vitro research. Some of these results might also suggest that ENPs can pose risks in the short-term and long-term. It is not entirely obvious how useful these statistics are for risk assessment. There are currently relatively limited data on the general public’s exposure to either chronic low-level exposure or acute high-dosage exposure of airborne ENPs. Acute high-dosage exposures are additionally improbable to occur. Regardless of any biological risk connected with ENPs, the risk from such exposures for negative CNS effects is most likely quite low [276,277,278].

## 7. Pharmaceutical Stability Testing of Nanomedicine

All NMs should be tested for temperature stability and moisture sensitivity under long-term and expedited transportation and storage settings. Higher temperatures are used in accelerated thermal stability testing to allow quick reactions extrapolated to the desired storage condition. In general, ICH standards propose long-term and accelerated storage settings of 25 ± 2 °C/60% relative humidity (RH) ± 5% RH and 40 ± 2 °C/75% RH ± 5% RH, respectively. According to the ICH standards, an intermediate storage setting (30 ± 2 °C/65% RH ± 5% RH) is recommended. Additionally, according to ICH recommendation Q1B, photostability testing is suggested for light-sensitive drug products. Furthermore, if any drug goods are intended for refrigerator storage, the ICH standards propose 5 °C, 3 °C, and 25 ± 2 °C/6% RH ± 5% RH for long-term and accelerated storage, respectively. Only the long-term storage setting of −20–5 °C is suggested if any drug goods are stored in a freezer. According to ICH recommendations, NMs should demonstrate physical, chemical, and microbiological properties as new medicinal products under appropriate thermal storage settings and durations [279].

## 8. Current Status of Nanomedicine

A wide range of NPs has been developed and tested in preclinical and clinical research. Polymeric NPs have a lot of potential in drug delivery, imaging, treatment, and theragnostic applications. Several novel NPs in the early stages of development have the potential to improve and facilitate cancer imaging and therapy. Polymeric NPs, such as biodegradable NPs, micelles, and stimuli-responsive NPs, have proliferated over the last decade due to their unique physical properties, including selective targeting, controlled release, and protection of deliverable agents, and increased circulation time in the body. Polymeric micelles provide new tools for loading poorly water-soluble anticancer drugs and increasing the molecules’ lifetime to develop novel therapeutic entities in cancer therapy. In vitro and in vivo, stimuli-responsive polymeric NPs have exceptional regulated release patterns, resulting in more potent anticancer effects. Polymeric NPs provide good contrast enhancement for practically all medical imaging modalities, allowing researchers to track NP kinetics to track tumor activity. NPS can also be used for MI theragnostics. If tumors have high NP accumulation, a burst release of the medicine can be achieved by introducing a physical source to the NPs, such as NIR lasers, external heat, or illuminating photosensitive components. NM’s future, mainly through polymeric NPs, will improve and facilitate traditional medicines to help humans personally and globally. Continuous research on polymeric NPs in preclinical and clinical trials will significantly alter and improve illness diagnosis, therapy, and prevention [280,281,282].

## 9. Challenges in the Physicochemical Characterization and Designing of Nano Formulation

Developing a new medicinal product is a systematic and orderly procedure that accumulates information from start to finish, commencing with research on the physicochemical qualities and biological activity and ending with “proof of concept” to prevent wasting resources and time. When presenting scientific information, there are various important factors to consider. Initial research should focus on the NF’s physicochemical characterization, production stability, regulatory issues, and agency recommendations. In vitro assays, such as cell lines, should be correlated with in vivo assays and selecting the appropriate tissue and animal model, considering the first elements of the medicinal product. Numerous issues have been highlighted by displaying the various systems that regulatory authorities have approved. The parameters and obstacles in obtaining NPs for biomedical applications are described in the following sections [283].

### 9.1. Physicochemical Characterization

Unlike the development of other therapeutic products, evaluating the toxicity potential of NPs in biological systems starts with a complete physicochemical characterization, which is a critical step in the early stages of development and contributes to the principles of quality, safety, and efficacy. It is vital to use proper characterization technologies that correlate impact and biological repercussions and forecast toxicological and therapeutic results early in the product development process to make a valuable product in the clinical context. NPs are dispersed throughout any organ or system, crossing epithelial and endothelial barriers, and even diffusing them into cell membranes via diverse methods. The toxicity of NPs is determined by examining their physical and chemical properties and their link to undesirable outcomes such as thrombosis and platelet aggregation. They can infiltrate cellular organelles (mitochondria, nucleus), causing metabolic disruption, DNA damage, and cell death. Functionalization is a method for altering the physicochemical properties of NPs that involves attaching molecules to the surface of the particles, such as folic acid, biotin, oligonucleotides, peptides, monoclonal antibodies, and functional groups. This modification enables precise incorporation or alteration of specific features in NPs. Non-invasive properties, anti-agglomeration, and good physical qualities characterize the functionalized particles. Covalent and non-covalent bonds can be used to bind things together. Non-covalent conjugation allows for changes to be made without changing the molecular structure. It is feasible to increase the solubility, biocompatibility, biodistribution, and clearance of NPs by changing their surface chemistry, hydrophobicity, or charge through functionalization. The absorption and biocompatibility of NPs have been primarily addressed through functionalization; nonetheless, the efficiency of their uptake, the particles’ cytotoxic effects on cells, and their ability to penetrate biological barriers limit the clinical utility of these systems [284,285].

The physicochemical features of NPs influence their cellular absorption (active or passive). Through functionalization, it is feasible to improve particle stability and minimize aggregation. In biocompatibility, changing the surface charge of NPs and inactivating chemical groups that disrupt the cell membrane has been demonstrated to enhance formulations. The inclusion of molecules such as human albumin, for example, can lower the particle’s toxicity. Sanità et al. revealed in 2020 that albumin functionalization on silver–eumelanin hybrid NPs reduced normal mammary cell chemotoxicity in vitro (MCF10a). Incomplete particle analysis is the primary reason for their approval for use in human treatments [96]. Size, shape, content, charge and surface chemistry, encapsulation efficiency, and evaluation of the encapsulated drug with factors such as loading, distribution, release kinetics, interaction with cells, and transport system should all be part of the characterization. There are few defined techniques for the physicochemical and biological characterization of NMs, and recommendations are needed to assess the quality and safety of these new products. For each of the NPS, new or updated methods for evaluation may be required. To decide the most proper selection, it is necessary to understand each approach’s pros and disadvantages that provide trustworthy information for a specific parameter.

From the start of the characterization, there are issues with selecting the best appropriate technique for determining the physicochemical parameters. Dynamic light scattering (DLS) is the most extensively used method for measuring the diameter, particle size distribution (PSD), and stability of NPs in suspension. At the nanoscale, this process has International Standards and Guidelines. However, the results are conflicting. It has problems monitoring minimum diameter variations and estimating the size distribution, and it only works if the particles are monodispersed. To analyze the size, PSD, and stability, it is advisable to integrate several methodologies in a complementary manner. Electron microscopy (EM), which offers images with higher contrast, a high atomic number of elements, and a resolution of less than 1 nm, is the most powerful tool for determining the morphology and size of NPs. Transmission electron microscopy (TEM), which allows a large population of particles to be observed with high lateral resolution; scanning electron microscopy (SEM), which determines topochemical data and surface defects; and a combination of both: transmission and scanning electron microscopy (STEM), which allows the size distribution, interfacial structure, compositional distribution, and surface defects to be determined. However, because these approaches analyze a small, non-representative sample, it is impossible to verify the presence of agglomerates, and it does not accurately identify the organic covering of individual particles. Another alternative to mass spectrometry (MS) is atomic force microscopy (AFM), which allows for a three-dimensional study of NPs, mapping surface heterogeneity and determining their thickness without sample preparation or electron beam exposure. The regulations recommend combining numerous procedures with varied configurations for regulatory objectives and properly evaluating morphology and size.

Encapsulation efficiency (EE), a qualitative factor that evaluates the drug loading capacity in the particles, is another essential measure in the characterization of NPs. It is determined by the preparation method, the drug’s physicochemical qualities, and formulation variables. The main challenge in calculating EE is the accuracy of the drug analysis, which is complicated by the use of both direct and indirect procedures, and a combination of both is preferable to obtain more accurate results (drug-loaded solid lipid NPs were separated by centrifugation before measuring the unencapsulated drug in an aqueous phase, in other studies, the drug content was directly measured to determine EE percent). The release kinetics of NFs is another important quantitative measure when evaluating their properties. This trait should be investigated by examining factors that can impact the process and determining how the drug is released (slowly and steadily or entirely). Although the medication loaded impacts the release pattern, this assessment is dependent on the composition of the encapsulating matrix. The points that change the formulation must be identified and evaluated, and the presentation of the NPs must be determined: suspension, gel, cream, tablets, aerosols, or NCPs. It has been shown that the zeta potential of the NPs influences the viscosity, stiffness, and gel network structure of gel NF (chitosan gel containing acrylic-based NCs) [284,285,286,287]. The thickness of the formulation can be affected by the encapsulation efficiency, shape, and diameter of the particles in the case of tablets. Particle-related parameters (size, distribution, morphology, zeta potential, composition, charge, and surface chemistry) and entrapment parameters (drug charge and distribution, encapsulation efficiency, release kinetics, and cell interaction) are considered complete characterization of NPs. It is critical to utilize the most appropriate procedures in each circumstance when evaluating these features.

### 9.2. Next-Generation Nanomedicines

#### 9.2.1. Stimuli-Responsive and/or Actively Targeted Nanomedicine

In the past two decades, considerable efforts have been devoted to creating a myriad of smart nanocarriers that enable precise function (e.g., tumor penetration, drug release, cellular uptake, and endosomal escape), relying on tissue micro environment and available exogenous stimuli [288].

##### Tissue Microenvironment-Responsive Nanomedicine

The responsiveness of NM (such as cleavage and protonation) is widely explored using pH as a common biological cue. Because the majority of NM may travel into endosomes or lysosomes, these organelles are the natural acidic reactors for intracellular delivery. For instance, to help proteins and genes escape from the endosome more effectively, we started a charge-conversion polyplex system based on pH-cleavable maleic acid amide derivatives.

##### Externally Triggerable Nanomedicine

Although intriguing and promising, tissue microenvironment-responsive NMs achieve elegance via biological cues, which usually exhibit heterogeneity. An alternative is to use external energy to directly interact with NM-retained tissue, such as light, magnetic field, and ultrasound.

##### Actively Targeted Nanomedicine

By adorning the surface with affinity ligands to activate receptors, actively targeted NM enables extended tissue retention and higher cellular uptake. High bioavailability may theoretically be anticipated. The most often utilized ligands include aptamers, antibodies, peptides, and small compounds. By increasing therapeutic efficacy through improved delivery to the locations of the disease, NM is a potential approach for enhancing clinical outcomes for cancer therapy. The aspects that go into reaching this aim, such as the size, chemistry, and functionality of NP delivery systems, as well as how these elements affect tumor localization and uptake, are crucial for eventual clinical success.

#### 9.2.2. Tissue Microenvironment-Reprogramming Strategies for Rejuvenating Nanomedicine

As time goes on, the diversity of pathophysiological characteristics provides the fascinating urge to create more active methods of reprogramming the tissue microenvironment to improve the performance of NM, particularly tissue accumulation and responsiveness. Increasing vascular permeability and perfusion, decreasing stroma, boosting receptor expression, and causing a rise in stimulus concentration are some of the enticing ways [289].

#### 9.2.3. Tissue Microenvironment-Reprogramming Strategies for Amplifying Tissue Accumulation

The majority of studies aimed at accelerating tissue accumulation emphasize upregulating receptor concentration and increasing vascular permeability/tissue perfusion. The decision of the tumor’s destiny depends critically on the tumor microenvironment (TME). Tumor cell proliferation, metastasis, immune evasion, stemness, and treatment resistance are all facilitated by TME components. Therefore, altering the TME to specifically target tumor cells might be an all-encompassing and successful anti-tumor therapy. Normal cells can enter and disturb the aberrant TME, including immune cells and stem cells. An intriguing new approach to treating tumors is the reconstruction of the TME using healthy cells.

#### 9.2.4. Tissue Microenvironment-Reprogramming Strategies for Amplifying Responsiveness

A fundamental biological premise for NM is that the microenvironment of sick tissue can substantially initiate a desired occurrence, such as medication release resulting from stimuli-responsive behavior. Based on our limited knowledge of the microenvironment and nanobio interaction, this is not always the case. Hazardous drug release or generation is a crucial factor in determining effectiveness. Unfortunately, these factors have not been well studied or often uncharacterized in vivo, despite the presence of a flawless release profile in vitro. As evidenced by some examples, such as NK102, CRLX101, and liposomal formulations containing doxorubicin, cisplatin, or CPT-11, the majority of (pro)drug is stubbornly trapped in the original formulation or not transformed into toxic constituents, despite apparent tissue accumulation even 0.7% of the administered dose from a meta-analysis study is still high compared with free agents.

### 9.3. Transcytosable Nanomedicine

To maintain tissue metabolism and homeostasis, a common transport pathway called transcytosis involves the vesicular translocation of biomacromolecules through epithelial or endothelial barriers. In addition to the dominant focus on paracellular transport, some work was completed to take advantage of the active transcellular mechanism known as transcytosis. For active luminal-to-abluminal transport in tumorand brain endothelium, NM is based on a number of transcytosis types, including receptor-mediated transcytosis (RMT), absorptive-mediated transcytosis (AMT), and bulk-phase or fluid-phase transcytosis (FPT).

### 9.4. Immuno-Oncological Nanomedicine

Most notably, immunotherapy has transformed oncological treatment following chemotherapy, radiation, and surgery, particularly in light of the efficacy of immunological check-point blocking (ICB) therapy (e.g., anti-PD-1/PD-L1 or anti-CTLA-4 antibodies). However, because tumor immune microenvironments (TIMEs) are varied and there are several methods to evade immune monitoring, only around 15% of patients might have an objective benefit. Additionally, immune-related adverse effects are common, occasionally serious, and occasionally even life-threatening. Precision medicine is made possible by patient classification, yet patients who are resistant to immunotherapy may still not benefit from it. In this situation, it is hoped that NM would increase both the safety and effectiveness of immunotherapy.

#### 9.4.1. Targeting Tumor Tissue

ICD may mature dendritic cells, improve their absorption of antigens, eventually activate T-cells, and produce damage-associated molecular patterns that secrete and produce antigens. As monotherapy or in conjunction with ICB, such as Doxil and nanoscale coordination polymers, ICD from NM-based chemotherapy, photodynamic treatment, hyperthermia therapy, and radiation can enable site-specific action, boost bioavailability, and minimize systemic toxicity. Epirubicin-conjugated micelles have recently been validated to increase the effectiveness of anti-PD-1 antibody treatment against glioblastoma, which is frequently resistant to ICB therapy.

#### 9.4.2. Targeting the Tumor-Extrinsic Immune System

More significantly, NM might be used to deliberately increase the effectiveness of therapies that target the tumor-extrinsic immune system (such as lymphoid organs, DC, T-cell, and immunosuppressive cells). Primarily, this complex intercommunication fosters the environment necessary for tumour cell growth, survival, and metastasis. It’s interesting to note that immune cells have a significant influence on both their prospective anti-cancer cellular arsenal and their opposing pro-tumoral function.

#### 9.4.3. Enhancement of Interaction between Immune and Tumor Cells

NM’s functionalization of various signaling molecules of different types might further enhance the interaction between immune and malignant cells. For instance, a liposome decorated with E-selectin and TRAIL demonstrated binding to blood lymphocytes via the E-selectin receptor, increasing the presentation of TRAIL to circulating tumor cells via death ligands, and causing cytocidal activity. By utilizing immune stimulators based on the immune cell’s capacity to actively look for tumor cells, this platform could become more effective. Another example is the use of polystyrene NPs tagged with anti-HER2 antibodies and the “eat me” signal calreticulin to specifically target cancer cells that are HER2-positive while simultaneously labeling the target cells with calreticulin to aid with APC processing of the antigen.

#### 9.4.4. Intracellular Delivery

The effectiveness of some immune stimulators, such as negatively charged nucleic acids, is restricted by the cellular absorption and endosomal escape barriers of cytosolic administration. Nanocarriers may facilitate the transport of stimulators into the cytosol. For example, the cyclic dinucleotide agonist of STING was created to be enclosed in endosomolytic polymersomes that self-assembled from block copolymers carrying membrane-destabilizing regions, facilitating its transport to the cytosol.

#### 9.4.5. Reprogramming Tumor Microenvironments

Additionally, restoring a normal vasculature and microenvironment may cause immunosuppressive tumors to develop immunostimulatory characteristics, in part because of rising immune cell infiltration, which is a promising subject for future research. If combined with altering the tumor microenvironment, this immune activation facilitated by NM could further enhance cancer immunity.

## 10. Socio-Environmental Considerations

Nanomaterials’ impact on the environment and public health has received a lot of attention. However, as NM and the broader area of NT advance, much more work remains to be completed, mainly because different nanomaterials pose different types of dangers. This section will concentrate on the NP kinds mentioned in the previous applications. In vivo investigations will be highlighted, as they have been in prior areas, but substantial in vitro work will also be discussed.

### 10.1. Toxicity and Safety Concerns

The small size of NF of pharmaceuticals allows the delivery of drugs made of inorganic materials into regions of the body that would otherwise be inaccessible due to their vast particle size. This is a massive benefit in medication delivery systems, raising concerns regarding unintended human body effects. NFs allow for lower anticancer drug doses, resulting in less toxicity, which can be further reduced by precisely delivering these drugs to tumors. A correct delivery technique allows for the maximum concentration of anticancer medicine within the tumor while avoiding high harmful levels of the drug in the surrounding normal tissues. Because the human body is not exposed to NMs, toxicity is not an issue when using NPs for in vitro diagnostics. In vivo diagnostic imaging with NP contrast material, on the other hand, should provide a safe and non-toxic dose. NPs’ biological effects are determined by their size, chemical composition, surface structure, solubility, shape, and aggregation. These variables can affect cellular absorption, protein binding, translocation from the portal of entry to the target site, and tissue injury risk. NPs’ effects depend on the routes of exposure, including the gastrointestinal tract, skin, lungs, and systemic delivery for diagnostic and therapeutic purposes. The biological impacts of NPs and their ability to spread throughout the body are influenced by their interactions with cells, bodily fluids, and proteins. NPs that bind to proteins may form more mobile complexes that can enter ordinarily inaccessible tissue locations. Accelerated protein denaturation or degradation can cause functional and structural alterations on the NP surface. Toxicologists have studied the effects of bigger particles created by implant wear and aerosolized particles of all sizes for several years. However, there is a scarcity of data on the health effects of nano-engineered particles smaller than 20 nm. Although larger NPs have been utilized to attack cancer cells, the critical worry will be particles less than 50 nm, which may quickly enter normal cells [290,291].

**Figure 12:**
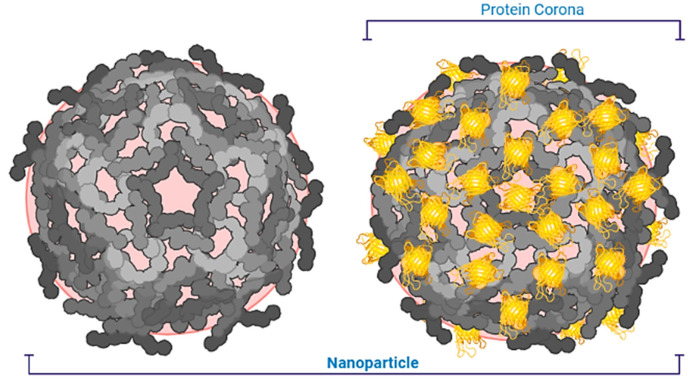
Protein corona formation by nanoparticles reduces the toxicity of NPs.

Many questions about their fate in the living body remain unresolved. Only 8% of the more than 150,000 publications dealing with NMs for various uses address their toxicity. Because of human exposure to NP-containing gadgets and manufacturing pollutants, there is worry regarding the toxicity of nonmedical applications of NMs. These impacts will vary significantly according to the extensive range of materials employed and the size range of NPs. Even if they are the same size, NPs of various materials have diverse properties, but the impacts of NPs of the same material vary in size. Even if they are the same size, NPs of different materials have various properties, but the implications of NPs of the same material vary in size. Some polymer NPs are biodegradable and cause less toxicity. Because NPs can cross the BBB and enter the brain after being introduced into the systemic circulation, concerns concerning neurotoxicity have arisen. The effect is dependent on the type of NP used, as some have even neuroprotective properties, such as cadmium telluride. Specific sizes of some materials may turn out to be poisonous. It is impossible to make a categorical statement about NP safety; in other words, it is impossible to tell whether NPs are entirely safe or dangerous. Each NM’s safety should be determined separately. Additional investigations, including those into environmental protection, are ongoing. Meanwhile, no significant side effects from the clinical usage of NMs have been reported [292,293]. Figure 12 dispatched the protein corona formation by NPs to reduce the toxicity of NPs.

### 10.2. Toxicity in In Vitro Models

**Figure 13:**
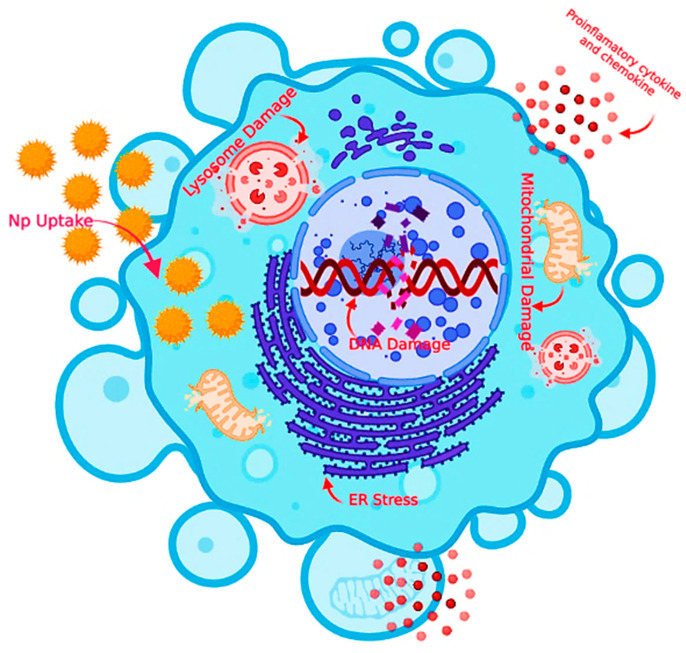
ROS generation and downstream processing in cells with response to nanoparticle uptake.

For formulation application, understanding the processes involved with nanotoxicity is critical. Cytokine generation, inflammatory stimulation, and increased reactive oxygen and nitrogen species, leading to apoptosis, necrosis, autophagy-mediated cell death pathways, and cytotoxicity, are some of the earliest harmful effects of NPs. The detrimental role of apoptosis in NPs toxicity was determined by measuring the formation of redox species (ROS) during mitochondrial dysfunction. Endothelial reticulum system (RES) stress, lysosomal dysfunction, and normal functioning are all harmed when mitochondrial dysfunction occurs during cellular rescue processes. NPs can trigger the inflammasome and extracellular trap formation in neutrophils, stimulate epigenetic alterations in macrophages to induce polarization and reprogramming, boost pro-inflammatory cytokine production, and activate the complement system. Non-oxidative pathways such as diminished nucleotide, carbohydrate, amino acid, and energy metabolism can also induce cell damage caused by NPs. Various approaches can be used to analyze cell viability to anticipate toxicity before animal testing, reducing the impact on living organisms [206]. The four primary approaches are loss of membrane integrity, loss of metabolic activity, loss of monolayer adherence, and cell cycle analysis. Monitoring and regulating ROS levels is critical, and the crucial role of NPs in ROS formation and its repercussions necessitates the assessment of nanotoxicity. Fluorescent Probes for ROS Measurement, Genetic Approaches for ROS Detection, Nanoprobes for ROS Detection, and Nanoelectrodes for Measurement of ROS in Superparamagnetic Iron Oxide NPs are some of the new and more accurate assays now available [294,295]. Figure 13 illustrates the ROS generation and downstream processing in cells in response to NP uptake.

in vitro models can be used to research immunotoxicity pharmacokinetics and immunological responses, while cytokine expression is best studied using enzyme-linked immunosorbent assays, flow cytometry, and reverse transcription-polymerase chain reaction. The entire toxicology cannot be analyzed due to the restricted in vitro approaches for predicting immunotoxicity. However, there are no specific regulatory procedures for measuring NP immunotoxicity in vitro. The same is true for carcinogenicity; many formulations are designed to be anti-carcinogenic, although cancer risk is considerable. Nanotherapeutic product research is equivocal, and the database for assessing carcinogenic risk, both qualitative and quantitative impacts, is lacking [292,293,296,297]. Because conventional animal models are insufficient to measure hepatotoxicity adequately, the liver is the organ of most significant concern and exploration when assessing organic toxicity for NPs. To summarize, the evaluation of these systems to identify the in vitro toxicity of formulations poses substantial challenges for in vitro assays due to the numerous discrepancies between in vitro and in vivo study designs. To construct the test in a cell system that is highly similar to human in vivo circumstances, it is required to look at the overall in vitro model, including the cell lines, exposure medium, culture system, and route of administration of the NF. More evidence is still needed to support the studies.

## 11. Preclinical Research for NPs

The evaluation of animal and human efficacy and safety information is a step in developing a pharmaceutical product. Preclinical toxicity assessment goals often include characterizing harmful effects on target organs, dose dependence, relationship to exposure, and reversibility. This data can be used to calculate a safe starting dose and dose range for human trials and identify clinical monitoring criteria for probable side effects. Early in the development of a formulation or drug candidate, safety research is required to gather the initial information on the product’s tolerance in various indication systems that will be important for future decisions. Preclinical toxicity assessment should be performed in two animal species with multiple doses and extensive treatment periods. Gender and age bias must be minimized when choosing an animal model, as they must be related to the time of disease start in humans to avoid data extrapolation failures. The route of administration influences the physicochemical characterization, in vitro, and in vivo characterization of the NF. The accumulation of the NPs system in organs, target tissues, and non-specific tissues and obstacles and environmental changes in the biological system should be used to choose candidates. An explanation of the route of administration aspects can be found in the FDA guidance for NPs in the industry. Inhalation administration of NPs, for example, should include accumulation/translocation, and subcutaneous delivery of NPs may increase sensitization to other allergens. Preclinical testing is difficult to standardize because the therapeutic index frequently affects drug dosage. NM dosing recommendations are based on excellent particle drug designs, NP characteristics, agglomeration states, and biodistribution data adjustments. The drug delivery dose is the patient’s administered amount (mg/kg body weight or surface area). For NPs, it can be expressed as the number of particles delivered; however, the amount of drug enclosed must always be taken into account for meaningful animal testing comparisons [298,299,300].

## 12. Nano Metal–Organic Framework

Metal–organic self-assembling nanoscale metal–organic frameworks (NMOFs) are a unique family of materials that combine the advantages of both bulk MOFs and nanomaterials. First, NMOFs may load imaging agents and/or medicinal preparations easily due to the very porous and wide channels. Meanwhile, by covalently attaching the construction units—whether metal ions/clusters or organic ligands—both can be employed to payload imaging payloads and/or medications. NMOFs-based diagnostic and therapeutic agents, such as those used for magnetic resonance imaging (MRI), computed tomography (CT), positron emission tomography (PET), and optical imaging, have been extensively developed and employed. Second, the right choice and functionalization of organic linkers and building blocks enable NMOFs to load and release drug and imaging agent components in controllable spatial and temporal patterns, highlighting their latent functions on theranostic platforms. For biomedical optical applications, NMOFs are a desirable contrast medium due to the inorganic metal components on them. Until now, NMOFs have been encapsulated with therapeutic building units for specific treatments. For example, employing photosensitizers as construction groups allows NMOFs to be successful for photodynamic treatment (PDT), while high-Z metal ions-containing NMOFs materials with outstanding X-ray attenuation ability might be utilized as a radiosensitizer to improve field radiotherapy (RT). Thirdly, it is possible to create activated or controlled-release nanocarriers thanks to NMOFs’ adaptable skeletal stability in physical environments. Additionally, a variety of NMOFs has received certification for minimal cytotoxicity, guaranteeing their use in vivo. The NMOFs are naturally biodegradable due to the much weaker metal-ligand coordination connections. The NMOFs are a viable platform for the biological industry because of their unique qualities [301].

Metal-organic frameworks (MOFs) are a rapidly expanding and interesting class of organic–inorganic hybrid materials that are made of inorganic metal ions or clusters and multipoint organic bridge linkers. The physical and chemical properties of MOFs could be rationally controlled and regulated for specific applications due to the variety of connections between organic linkers and metal ions, particularly when the size of MOFs is reduced to the nanoscale regime, which makes it a leading platform in biomedicine. Among hybrid nanomaterials, nanoscale MOFs (NMOFs) is a distinct class. NMOFs are excellent candidates for use in biological sciences because they have the same characteristics and structural diversity as bulk MOFs while keeping their size at the nanoscale level. Additionally, NMOFs offer a number of potential benefits over currently available nanocarriers, which gives them a larger range of possible applications in biomedicine: The following factors make it possible to create NMOFs with a variety of constitutions, shapes, and characteristics: (1) structural and compositional adaptability; (2) oriented frameworks and high porosity provide space for latent therapeutic agents and different imaging efficiency; and (3) weaker coordination bonds make them easily biodegradable [302].

Scaling down particle size inside the nanoscale should receive priority consideration for biological applications. NMOFs are porous nanomaterials that can include an infinite number of inorganic and organic components. Two things should often be taken into account when purchasing nanoscale MOFs: limiting supramolecular assembly, which often results in crystal development at nanoscopic scales, and promoting nucleation against crystal growth, are two examples of this. The four types of controlled synthetic methods for NMOFs that are currently covered in the literature include reverse microemulsion, surfactant-assisted approach, solvo-thermal synthesis, and nanoprecipitation. The development and nucleation of NMOFs during the synthetic process in these techniques are influenced by variables such as reactant ratio, temperature, pH, reaction conditions, and others. In comparison to conventional NMs, NMOFs have a number of advantages, including structural variety, loading capacity, and biodegradability. Many NMOFs have received certification as bioimaging agents for use both in vivo and in vitro. To create NMOF-based NM, researchers have employed two general strategies: silica coatings or organic polymers are used to modify the surface of the NMOFs after they have been synthesized to increase their stability, slightly regulate their physicochemical characteristics, and provide additional functionality and biocompatibility. The first strategy involves adding active groups to the skeleton of the NMOFs or loading active agents into their channels and pores. For the pore encapsulation technique, NMOFs as a host material can stop the loaded substrates from leaching as well as give them a safe haven from harmful outside influences. It is crucial in the clinic that the surface functionalization technique for NMOFs can gain the desired function and adhere to the strict specifications. These two methods, however, need stable NMOFs and substrates under synthetic circumstances [303,304].

## 13. Future Prospects

The initial excitement around NM was dampened by worries regarding NP safety, many of which have already been addressed, and additional research is ongoing. Since its inception a decade ago, this publication has published ground-breaking research in various NM-related fields. As nanopharmaceuticals and devices are translated into clinical applications, technical breakthroughs in NB, like other fields of biotechnology, have posed safety and ethical challenges for physicians, scientists, and the general public. Until regulatory bodies develop unique procedures, medications utilizing NPs and drug delivery techniques are controlled in the same way as any other biopharmaceutical product. There is a discussion regarding whether NM requires unique rules because they must be tested for efficacy and safety before being used in humans. The US Food and Drug Administration classify new products as medicines, devices, or biologics. However, NB mixed with biologics such as stem cells may blur the lines between these categories. There is a need for a public impact assessment of NB, which was previously used primarily in engineering, communications, electronics, and consumer products. Still, now that it is being used in pharmaceuticals, food sciences, and human medicine, there is a greater awareness of the consequences for consumers, some of which are still unknown.

## 14. Future Perspectives Based on Pharmacokinetic Properties

To assess toxicity, pharmacokinetic investigations of NF are also required. There are few pharmacokinetic studies on NPs, and there are no recommendations for NMs, making it difficult to assess this parameter. Absorption, distribution, metabolism, and excretion are the four processes that makeup PK. The area under the curve (AUC), clearance (CL), the volume of distribution (VL), mean elimination time (t_1/2_), maximum plasma concentration (Cmax), renal clearance (CL), and the mononuclear phagocyte system are all measured in each case. The rate and degree of absorption are influenced by the physiological environment and the NPs’ characteristics. Nano formulations pass across physiological and physical barriers that selectively block the transport of molecules, reducing NP bioavailability. Cellular uptake is heavily influenced by size, surface charge, and shape. The route of administration and the characteristics of the NPs affect absorption. UV radiation absorption rate, penetration, size, and effect on the dermis should all be examined. The negative surface charge has a higher absorption rate at the gastrointestinal membrane in the oral route, and it is related to the size of the small intestine. The pulmonary route has a larger contact area, which makes absorption easier. Biodistribution is influenced by physicochemical qualities (interaction with biological barriers and proteins), as well as composition (silica, polymers, proteins, metals, lipids), size, shape, surface charge, and hydrophobicity. The PK of biopharmaceuticals containing NPs is more complicated; “nanometric and non-nano forms” persist in the biological media after administration and absorption.

Given the requirements for NM development and use, assessing the nature of formulations, pharmacokinetic qualities, and the NM approval procedure are necessary steps to achieve clinical significance. As a result, we present a simple approach to guide the recommended ADME analyses of NMs based on contemporary developments in NM research and FDA and EMA standards. The suggested algorithm determines whether ADME features of the medications of interest are appraised or not based on manufacturing process stability and simulated human settings. The assessment is different depending on the administration method and distribution. For example, whether orally delivered NMs are discovered in nano or non-nano forms in the gastrointestinal tract affect evaluation. As a result, the proposed method provides crucial and practical checkpoints in creating and assessing NM.

## 15. Challenges in the Clinical Translation of Nanomedicines

The process of translating NMs into the clinical setting is expensive and time-consuming. When compared to traditional formulation technology, which uses free medication dispersion in a base, NM technology is typically far more sophisticated (e.g., tablets, capsules, and injections). The biological hurdles, large-scale production, biocompatibility and safety, intellectual property (IP), government restrictions, and overall cost-effectiveness in contrast to present medicines are important concerns in the clinical development of NMs. Whether or if NMs are therapeutically effective, these issues can place considerable barriers in the way of their commercialization.

### 15.1. Biological Challenges

NM development has historically been predicated on a formulation-driven strategy, whereby new delivery systems are first developed and characterized from a physicochemical perspective. Limitations in the clinical translation of the system have just recently been discovered when seeking to match the NM with a pathological application. Understanding the interplay between biology and technology is crucial for the successful translation of NMs. This includes comprehending how disease pathophysiology affects the accumulation, distribution, retention, and efficacy of NMs as well as the biopharmaceutical correlation between delivery system properties and in vivo behavior in animals versus humans. In order to enhance clinical translation, it has been proposed to use a disease-driven strategy by creating NMs that may take advantage of pathophysiological changes in disease biology.

The relationship between disease pathophysiology and the heterogeneity of the disease in humans, as well as the significance of the physicochemical characteristics of various NMs to overcoming biological barriers to enable improved targeting of diseased tissue and/or reduced accumulation in non-target organs, must be taken into account from the beginning of NM development. Less study has been completed to fully understand the relationships between patient biology and NM behavior in particular therapeutic applications, as well as patient illness heterogeneity, which are likely the main causes of the failure encountered in the translation of promising NMs in clinical trials. The pharmaceutical industry’s investment in NMs may be seriously hampered by these biological difficulties. Preclinical data sets must thoroughly assess treatment effectiveness, safety, biodistribution, and pharmacokinetics in pertinent animal models of the disease that are representative of human disease in order to lower investment risk for NMs. To establish consistency of results for the specific illness and not for a single animal model, it is preferable to evaluate NMs in a variety of preclinical animal models that replicate characteristics of the clinical disease. Additionally, animal models that only represent a small portion of the clinical condition may offer insightful information about their potential for treating a particular patient subgroup. Depending on the various methods of administration, it is important to consider how the anatomy and/or physiology of the animal species differ from that of humans. Preclinical research on NMs should be performed with adequate blinding and randomization to decrease bias. It should also be assessed against relevant controls, such as the gold standard of care rather than merely free medication. Much published research presently lacks these elements, which makes it challenging to evaluate clinical applicability and translatability. The design of preclinical studies to optimize NM performance in vivo, dosing regimens, and treatment combinations based on the particular clinical disease, as well as comprehension of the impact of disease progression and severity on NM performance, are additional factors to take into account. This will establish if some patient sub-groups could react to NM-based therapy more positively.

It is interesting to note that over 80% of articles on NM from only the past two decades have focused on cancer targeting, as have the bulk of NM formulations now in development and in clinical trials. The conversion of published findings to therapeutic applications has been poor, notwithstanding the high quantity of publications. Despite the fact that EPR-mediated accumulation has only been identified for a few tumor types, the EPR effect has traditionally been the basis for all cancer targeting of NMs. Similar to other clinical illnesses, tumors can exhibit both intra-patient and inter-patient variability as the condition worsens. Therefore, it is doubtful that a one-size-fits-all strategy would result in therapeutically advantageous outcomes for developing NM-based treatments. Other non-cancer illnesses, particularly those with an inflammatory component that induces leakiness of inflamed blood vessels, have increasingly taken advantage of the EPR effect for NM targeting (e.g., rheumatoid arthritis, atherosclerosis, and inflammatory bowel disease). Due to biological restrictions, not all diseases may be treated with NMs, and not all clinical conditions are expected to exhibit the EPR effect. The effectiveness of NM is not solely determined by EPR. The degree of cellular absorption and drug release kinetics inside target tissues both have an impact on NM activity.

Aside from that, despite the increased accumulation at target regions and therapeutic results in a number of preclinical trials, the benefits of ligand-targeted NMs in the clinical research phase have so far been minimal. The accessibility and expression of the target, disease-dependent anatomical and physiological obstacles, formulation stability, and other elements have all been discussed as potential causes of this disparity. Additionally, each NM’s surface must have the ideal targeting ligand density, which will probably rely on the properties of the molecular target (e.g., expression, location, internalization rate, and immunogenicity). Clinical validation and translation will also require careful assessment of the level of NM accumulation, cellular internalization, intracellular functioning, and intracellular degradation. Our knowledge of the more suitable therapeutic uses for ligand-targeted NMs is improving thanks to intensive testing. Thus, by approaching NM development from a disease-driven perspective, it will be feasible to create extensive preclinical data sets that can assist transferable clinical development and better predict efficacy for patient sub-groups.

### 15.2. Large-Scale Manufacturing

The formulation’s inherent structural and physicochemical complexity is one of the significant issues slowing down the clinical translation of NMs. Platforms that need time-consuming and/or complicated synthesis techniques often have little promise for clinical translation since large-scale pharmaceutical manufacturing can be very difficult for them. The advancement of pharmaceutical production is focused on both quality and affordability. Production of NM is hampered by possible problems linked to quality, which also covers the manufacturing process and formulation stability: (i) poor quality control; (ii) scalability complexities; (iii) incomplete purification from contaminants (e.g., by-products and starting materials); (iv) high material and/or manufacturing costs; (v) low production yield; (vi) insufficient batch-to-batch reproducibility, consistency and storage stability of the final product (e.g., regarding size distribution, porosity, charge, and mass); (vii) lack of infrastructure and/or in-house expertise; (viii) chemical instability or denaturation of the encapsulated compound during the manufacturing process; and (ix) scarcity of venture funds and pharmaceutical industry investment.

Having access to a preparation process that enables the creation of sizable amounts of NMs that are also routinely generated at a high degree of quality and batch-to-batch repeatability to established parameters is a crucial condition for clinical translation. Without the need for several manufacturing processes or the use of organic solvents, suitable techniques for the industrial-scale manufacture of certain fundamental NM platforms, such as liposomes, have been created successfully. The difficulties appear when the NM system gets increasingly intricate. For instance, adding surface modification through coatings and/or ligands, including numerous targeting elements, or encapsulating several therapeutic agents are some examples. Multiple production steps are necessary to integrate multiple components into a single nanosized carrier, which inevitably complicates large-scale good manufacturing (cGMP) production, raises production costs, and makes it more challenging to evaluate such products for quality assurance and quality control (QA and QC).

To begin clinical translation, the manufactured NM’s characteristics must be well specified and consistently produced. For investigational novel medicines (IND), information on chemistry, manufacturing, and controls (CMC) is necessary at every stage of the inquiry to guarantee the correct identification, strength or potency, quality, and purity of the drug material and drug product. The research’s stage, the scope of the human study, the length of the investigation, the nature and origin of the drug ingredient, and the dosage form of the drug product will all influence the kind of information that must be supplied. The sheer number of characteristics that need to be considered for the characterization and validation of increasingly complicated NMs (such as size distribution, shape, charge, purity, drug encapsulation efficiency, coating efficiency, and density of conjugated ligand/s) might be particularly difficult. NMs’ physicochemical characteristics, such as polarity and size, pharmacokinetic factors, such as absorption, distribution, metabolism, and excretion, as well as pharmacodynamic interactions, may all differ significantly from batch to batch (e.g., cellular interaction and activity). NMs must also be stable throughout the production process, for long-term storage, and during clinical administration (i.e., to avoid massive drug release or aggregation in the bloodstream en route to the site of action).

### 15.3. Biocompatibility and Safety

To assess the overall safety of NMs for human usage, detailed toxicology is crucial for clinical translation. At any stage of clinical development, pharmaceutical regulatory authorities typically advise the sponsor to carefully examine any changes to the drug substance, the manufacturing process for the drug product, or the formulation of the drug product to see if the changes could either directly or indirectly affect the product’s safety. Modifications to CMC made throughout the IND process that may have an impact on safety include: (i) modifications that result in a different impurity profile; (ii) modifications that affect the actual manufacturing process (such as chemical synthesis, fermentation, or derivation from a natural source); (iii) modifications that affect the source material; (iv) modifications that affect the method of sterilization of the drug substance or drug product; (v) modifications that affect the route of administration; and (vi) modifications that affect the dosage form (e.g., dose delivery).Stringent processes are in place to guarantee that, should any modifications be found, the drug substance and/or drug product generated using the old manufacturing process and the new manufacturing process are appropriately compared to assess product equivalence, quality, and safety. Sponsors should carry out extra qualifying and/or bridging studies to establish the safety and bioavailability of the substance to be used in the proposed trials where analytical results show that the materials prepared before and after are not similar.

Predicting potential side effects or toxicities in vivo requires knowledge of the activity and toxicities of the free drug, the behavior of various NM delivery systems and their interactions with biological components, and the influence of drug release rate on target and off-target concentrations of the bioavailable drug. To improve their ability to be clinically translated, NMs’ rational design from the early stages of material selection, production technique optimization, and product purification are particularly crucial. Even though the safety of some common substances, such as phospholipids and biodegradable polymers, has been previously researched, adding complexity to NMs, such as using various synthetic compositions, coatings, and ligands, can significantly affect the biocompatibility, biodistribution, and toxicology profile of NMs after in vivo administration. For instance, several NMs might elicit complement activation-related pseudoallergy (CARPA), an acute unfavorable immunological response. The innate immune response’s complement system participates in a variety of inflammatory and immunological events. The symptoms of CARPA, an acute, non-IgE-mediated hypersensitivity reaction, include anaphylaxis, facial edema, face flushing, chills, headache, and cardiac distress.Standard allergy medicines are usually used in conjunction with decreasing the infusion rate or stopping the treatment to control this adverse response (e.g., antihistamines, corticosteroids, and epinephrine). Immunogenic responses to NM-based treatments might change pharmacokinetics, reduce effectiveness, and increase the risk of potentially harmful toxicities (e.g., anaphylaxis).

In order to properly examine the nanotoxicology of NMs during the early phases of clinical development, there is a regulatory need for tests that are validated, sensitive, and standardize and that incorporate in vitro, *ex vivo*, and in vivo methodologies. Before doing preclinical testing on animals, comprehensive in vitro or ex vivo tests for nanosafety testing are necessary to identify any possible risks. To analyze the possible danger of cytotoxicity, immunotoxicity, and genotoxicity of NMs, for instance, standardized in vitro techniques utilizing various cell culture models (such as blood, liver, lung, brain, placenta, and gastrointestinal system) have been developed. With the development of NMs including several novel materials with the intention of usage in the therapeutic context, this is especially crucial. Standardized reference materials would need to be developed in order to do this across the board, and the testing would also need to be pertinent to the desired route of administration. Although there are a variety of procedures being developed that are better tailored for NMs, present testing methods for nanotoxicology evaluations for clinical translation remain constrained and inadequate. Alternative test methodologies, high-throughput screening methods, high-content screening, and computational modeling are all included in this. Many NMs might potentially be analyzed simultaneously using these approaches in a comparative manner.

As circulation half-lives and drug retention durations are typically greatly extended by nanoencapsulation, it is also necessary to conduct specific toxicology studies in animal models to evaluate both short- and long-term toxicity. To accurately anticipate the toxicological reactions to NMs, it is crucial to have a complete understanding of the absorption, distribution, metabolism, and excretion of developing nanomaterials in vivo. There is a need for adequate evaluation methodologies to track the pharmacokinetics, biodistribution, target site accumulation, local distribution at the target site, localization in healthy tissues, the kinetics of drug release, and therapeutic effectiveness of the NM drug delivery mechanism. Real-time imaging tools have made it possible to better understand how NMs interact with biological organs and tissues after being administered in vivo.

The potential for immunotoxicity, inflammation, and biocompatibility should also be evaluated, and the functional results should be connected with the processes of tissue absorption and clearance. Prior to clinical trials, these factors need to be thoroughly examined depending on dose, dosage form, and mode of administration to define safe limits. This is especially crucial for NMs made of materials that have never been utilized in clinical settings before. Regulatory measures should be in place even during the clinical trial phase to identify any toxicity brought on not just by the medicinal drugs they are encapsulating but also by innovative nanotechnology-specific mechanisms. For instance, the immediate and long-term consequences of NM accumulation in the organs that store the most NM after systemic delivery (liver, kidneys, spleen, lungs, lymph nodes, and bone marrow). Because the RES cells are also a component of the innate immune system, there have been questions over whether NMs’ saturation of macrophages might impair immunity and make people more susceptible to infections. NMs, despite claims that excessive NM deposition in macrophages can reduce their capacity for phagocytosis or alter other cellular processes. However, after absorption, NMs containing cytotoxic chemicals can cause the death of macrophages, and indirect signals may point to partial immune suppression. Due to the inhibition of macrophages caused by the administration of Doxil^®^ to mice, it has been observed that the removal of germs from the blood is hampered. To protect the use of new NMs in the therapeutic context, these challenges must be addressed.

### 15.4. Intellectual Property (IP)

More exact criteria of what constitutes the innovative IP of NM are required, given the difficulties in integrating nanotechnology into biological and clinical applications. NMs are complicated because they cross the gap between the fields of medicine and medical devices and involve a lot of changeable components. An NM product must typically have an IP position on the following three things: (i) the enclosed cargo; (ii) the carrier technology; and (iii) the features of the medication and carrier taken together. Although this definition is simple, it does bring up a few issues with the granting of patents up to this point. For instance, NMs that combine known medications with novel carriers or those that combine them with current carriers for a fresh biological or clinical application. More complicated drug delivery systems, such as those that use commercially available targeting ligands (such as antibodies) or coatings (such as Eudragit^®^), which are controlled by other firms, further complicate the IP position. Multiple patents linked to any particular technology may be a part of the IP strategy, as well as the requirement for cross-licensing agreements. In order to shorten the time and cost involved in negotiating cooperation and licensing agreements, new IP practices and standards are needed to streamline the process from invention to commercialization.

Other critical concerns that need to be addressed because of the huge growth in the number of nanotechnology patent applications over the past few decades include patent review delays, patent thickets, and the issuing of invalid patents. To prevent the issuance of several nanopatents on the same innovation, there has to be a universal nano-nomenclature on identical or comparable nanostructures or nanomaterials, as well as more sophisticated search tools and commercial databases. The Patent and Trademark Office’s (PTO) databases need to be able to search through nanotechnology-related prior art that was published in scientific journals all over the world, including earlier journals that were published before the development of online publishing databases. Examiners of patents also need knowledge of and training in the burgeoning industries of nanotechnology and NM. The so-called “patent thickets,” which can result in expensive litigation and stall commercialization attempts, are the result of the complexity of nanotechnology. Improved IP and patenting clarity surrounding nanotechnology in health and medicine is thus necessary, and this will necessitate the establishment of global laws and policies that are specifically designed for this specialized sector of commercialization.

### 15.5. Government Regulations

However, the existing scientific and regulatory gap for NMs is enormous and difficult. NMs have a big potential to accelerate the growth of the pharmaceutical sector and improve health benefits. The ability to commercialize NMs is strongly influenced by a variety of regulatory aspects based on governmental regulations about manufacturing procedures, quality assurance, safety, and patent protection. The development of NM products toward efficient and rapid clinical translation has been hampered by the absence of defined regulatory and safety criteria. The safety and effectiveness of polymers, for instance, are greatly influenced by their molecular weight, polydispersity, molecular structure, and conjugation chemistry. Polymers have been extensively studied as an efficient platform for NM methods. There is an urgent need for a suitable regulatory framework to aid in evaluation given the rise in innovative polymeric materials and complicated polymeric-based NM formulations. Given that each polymer-based NM is unique, it is crucial to take into account each one separately based on the dosages, administration methods, frequency of dosing, and intended therapeutic usage. The majority of other NM systems would share this.

The primary regulatory body of each nation now oversees the regulation of NMs within the established framework (e.g., FDA, TGA, and EMA). Despite the fact that NMs have been on the market for close to 20 years, the initial generation of NM products only needed to comply with minimum requirements in order to receive regulatory clearance as medical substances. The quality, safety, and effectiveness of NMs for clinical application cannot be confirmed by these rules today. This is due to several factors, including the NMs’ complicated structure, their ambiguous interaction with human cells and tissues, the intricacy of clinical application, and the multifunctionality of some formulations (e.g., integration of therapeutics with imaging diagnostics). It is necessary to bridge the gap between medical device and medication regulations using regulatory standards and methods that have been particularly proven for NPs. An NM’s complexity, mode of administration, pharmacokinetics, pharmacodynamics, and safety profile should all be taken into account, and information on the best clinical trial design and patient selection should also be included. The development of innovative products in the market can be hampered by excessive regulation, which can drive up the costs of obtaining regulatory approval and/or eat up a significant portion of a patent’s lifespan. A delicate balance must be struck between ensuring the safety and quality of NMs and avoiding over-regulation.

Global regulatory norms for NMs should be developed in conjunction with important nations with vested interests. Greater collaboration between regulatory authorities, academia, research, and industry is required, despite the fact that significant advances were accomplished last year. This is particularly significant because there are not many contract manufacturing companies in the world that produce NM goods in compliance with the standards for good manufacturing practice (GMP). It should be highlighted that this small group of manufacturing companies might be further split according to how well-equipped they are to produce particular NM platforms (e.g., liposomes, polymeric NPs, dendrimers, and drug–polymer conjugates). NMs created at these production facilities should be governed by the same regulatory requirements since it is possible that they will be commercialized in several nations. It will be necessary to thoroughly assess and record the manufacturing procedures for NMs, taking into account the right industrial standards for both quality assurance and the avoidance of environmental problems. The usual pharmaceutical requirements for purity, sterility, stability, manufacturing practices, and related industrial control standards will still apply to manufacture NMs. The evaluation of important NM physical properties, including as particle size and size distribution, surface chemistry, morphology, surface area, surface coating, hydrophilicity, porosity, and surface charge density, will also need the implementation of new analytical techniques and established methodologies. These approaches will change depending on the nanomaterials and nanostructures involved. In order to assure the effectiveness and safety of present and new NMs, regulatory authorities should collaborate to create testing methodologies and suitable standardized procedures for toxicity studies and regulatory requirements.

## 16. Conclusions

There have been significant advancements in NM over the last ten years. To fully achieve the potential of NM, additional effort must be made over the next 5 to 10 years, building on current advances. First and foremost, the future of NM will be determined by the rational design of NMs, and instruments based on a deep understanding of biological and medical processes. The type, developmental stage, and location of the disease, for example, may influence drug carrier design and targeting tactics. Second, more complicated systems, such as multifunctional NPs capable of targeting, imaging, and therapy, will be investigated in the future. Third, toxicity issues are of particular importance; fundamental research must be conducted to solve these difficulties if these technologies are applied successfully and efficiently. Furthermore, because the field’s highly interdisciplinary nature remains a crucial obstacle, the best course of action will be to teach a new generation of really interdisciplinary investigators with a solid multidisciplinary foundation. In conclusion, while NM is still in its early stages, these practical uses show great promise. NM’s impact on drug delivery is undeniable. Although the product candidates presented in this study are not the only NM products now undergoing clinical trials, they appropriately represent the range of NPs employed in medication delivery. Furthermore, this analysis ignores the dozens of additional products in the early stages of development. The list of NM-enabled goods will continue to grow in the future with an adequate evaluation of potential NP toxicities and further NT breakthroughs connected to complicated diseases such as malignancies.

## Figures and Tables

**Table 1:** Nanoparticles and their biological effects.

S. No.	Nanoparticle Studied	Cell Type	Functions/Outcomes	References
1	Iron oxide NPs	Myocardium from mice	Oxidant by Nox 4 overexpression	[6]
2	WO_3_-Pt NPs	Tumor cells	Oxidant. NADPH oxidase biomimetic	[7]
3	Silver NPs	Human umbilical vein endothelial cells	Oxidant by increasing Nox 4 expression	[8]
4	Silver NPs	Myocardium from rats	Increase in hypertension due to a decrease in NO levels	[9]
5	PLGA NPs	Hypertensive rats	Carrier. Treatment for hypertension	[10]
6	PLGA NPs	Human umbilical vascular endothelial cells	ROS scavenger at the vascular level and endothelial protector	[11]
7	PLGA NPs	Rat focal cerebral ischemia/reperfusion injury	SOD carrier	[12]
8	PEGylated quantum dots	AT1R-expressing cells	Carrier of angiotensin-II	[13]
9	Silica NPs	Human endothelial cells	Endothelial injury induced by mitochondrial dysfunction	[14]
10	Silica NPs	Aorta from rat	Endothelial dysfunction induced by oxidative stress	[15]
11	PVAX	Hindlimb and liver from an ischemia/reperfusion model in mice	Antioxidant, anti-inflammatory, and anti-apoptotic activity	[16]
12	PLGA-β-PEG-TPP NPs	Mitochondria-acting therapeutics	Nanocarriers	[17]
13	RNP	A middle cerebral artery from rats with cerebral ischemia/reperfusion injury	Neuroprotective agent due to its ability to scavenge free radicals	[18]
14	Redox-polymer nanotherapeutics	Brain from SAMP8 mice	Treatment of the neurodegenerative diseases	[19]
15	Nanoceria	PC12 neuronal-like cells	SOD and catalase mimetic	[20]
16	Nanoceria	Mouse hippocampal brain slice model of ischemia	Reduction of oxidative and nitrosative damage after stroke	[21]
17	Nanoceria	Murine macrophages	Anti-inflammatory and NO scavenger	[22]
18	Nanoceria	Cultured rat H9c2 cardiomyocytes	Antioxidant	[23]
19	Nanoceria	Murine myocardium	Antioxidant and anti-inflammatory	[24]
20	Nanoceria	Human aortic endothelial cells	Inflammatory effect	[25]
21	Nanoceria	Aorta from mice	Vascular dysfunction	[26]
22	Nanoceria	Arterioles from hypertensive rats	Vascular antioxidant	[27]
23	Nanoceria	Arteriola from rats	Prooxidant. Microvascular dysfunction	[28]
24	Iron oxide, yttrium oxide, cerium oxide, zinc oxide	Human vascular endothelial cell line	Pro-inflammatory	[29]
25	NPs based on polyoxalate	Doxorubicin-treated mice heart	Antioxidant and anti-inflammatory	[30]
26	Polyketal particles	Rat myocardium	SOD carrier	[31]
27	Silver NPs	Human pulmonary epithelial cell line 16HBE14	Dose and process of uptake	[32]
28	Silver NPs	Human alveolar epithelial cells (A549)	Spherical particles had no effect than silver wires	[33]
29	Silver NPs	Human alveolar epithelial cells (A549)	Cells were only sensitive to high Ag-ion concentrations	[34]
30	Silver NPs	T84 cells (ATCC CCL-248™), a human colorectal carcinoma cell line	Small AgNPs have significant effects on intestinal permeability	[35]
31	Silver NPs	Porcine kidney (Pk15) cells	AgNPs had only insignificant toxicity at concentrations lower than 25 mg/L, whereas Ag^+^ exhibited a significant decrease in cell viability at higher concentration	[36]
32	Silver NPs	Human HCE-T corneal epithelial cells	Mammalian cell toxicity was observed at high (8–12 μM silver ion) silver levels in serum-free culture	[37]
33	Silver NPs	RAW264.7 macrophages	Low cell pro-inflammatory cytokine activation was observed	[37]
34	Silver NPs	Human tongue squamous carcinoma SCC-25	Reduced proliferation and viability	[38]
35	Silver NPs	Alveolar epithelial cells, macrophages, and dendritic cells	Adverse effects were also only found at high silver concentrations	[39]
36	Silver NPs	Human microvascular endothelial cells	Loss of membrane integrity at higher concentrations	[40]
37	Silver NPs	Bovine retinal endothelial cells	Enhanced apoptosis	[41]
38	Silver NPs	Dalton’s lymphoma ascites	Anti-tumor activity	[42]
39	Silver NPs	HepG2 cells	Non-cytotoxic doses induced p38 MAPK pathway activation and led to the promotion of HepG2 cell proliferation	[43]
40	Silver NPs	HaCaT cells	HaCaT cells were found to be resistant	[44]
41	Silver NPs	HeLa cells	HeLa cells were found to be more sensitive	[44]
42	Silver NPs	Embryonic neural stem cells	Ag-NPs-induced neurotoxicity	[45]
43	Silver NPs	Primary mixed neural cell cultures	Strong effects of SNP associated with calcium dysregulation and ROS formation in primary neural cells	[46]
44	Silver NPs	Mouse brain neural cells	AgNPs could alter gene and protein expressions of β-amyloid (Aβ) deposition	[47]
45	Silver NPs	Human embryonic neural precursor Cell	AgNPs exposure causes a significant stress response in the growing Human neural progenitor cells (hNPC)	[48]
46	Silver NPs	HT22 mouse hippocampal neuronal cells	AgNPs modulated HT22 cell cycle, proliferation, induced oxidative stress, and 53BP1 recruitment	[49]

**Table 2:** Some bio-imaging applications of ZnO NPs.

Type of Material	Model	Reference
ZnO-NPs	Human skin and rat liver cells	[111]
ZnO-NPs	Skin tissue/cellular architecture	[112]
ZnO-NPs	Skin	[113]
ZnO-NPs	Blood cells of zebrafish; roots and shoots of *Arabidopsis* plants	[114]
ZnO-NPs	Plants tissues cell implosion	[115]
ZnO Nanocrystals	KB cells	[116]
ZnO@silica-NPs	Hela cells	[117]
CdSe(S)/ZnO-QDs	*S. oneidensis*	[118]
ZnO/Au@PEG-NPs	B16F10 cells	[119]

**Table 3:** Advantages of nanobodies relevant to developing therapeutics.

1	Highly stable and able to bind antigens with nanomolar affinity
2	High target specificity and lower inherent toxicity
3	It can be humanized
4	Combine the advantage of conventional antibodies with essential features of small molecule drugs
5	Able to address therapeutic targets not easily recognized by conventional antibodies (e.g., the active site of enzyme)
6	Administrated by routes other than intravenous administration
7	Produced cost-effectively on a large scale
8	Extremely lower immunogenic potential
9	Ability to cross the human blood–brain barrier to reach targets in the brain

**Table 4:** Summary of current nanoparticle technologies in medicine.

Area	Nanoparticle Type	Major In Vivo Applications	Significant Characteristics
Optical imaging	Quantum dots	Site-specific imaging invivo	Imaging of lymph nodes, lung blood vessels, and tumors. Greater intensity and resistance to photobleaching compared with conventional methods. Site-specific targeting via surface functionalization. Subcutaneous imaging without surgical incisions.
MRI	Superparamagnetic iron oxide NPs	Cancer detection	Enhanced contrast for imaging the liver, lymph nodes, and bone marrow. Paramagnetic properties can alter magnetic resonance relaxation times of selected regions or fluids in vivo.
Drug and gene delivery	Polymer- and liposome-based NPs	Cancer therapy	Targeted delivery by surface functionalization. Strategies for prolonging residence times in vivo (e.g., PEG attachment). Strategies for solubilizing water-insoluble drugs (e.g., paclitaxel). Multi-layer and multi-functional (e.g., chemotherapeutic and anti-angiogenic).
		Neurodegenerative disease therapy	Transport across the blood–brain barrier (e.g., by PEG incorporation). Superior to direct drug administration. Therapies for diseases unresponsive to small molecule drugs (gene therapy).
		HIV/AIDS therapy	Solubilizing water-insoluble drugs by emulsification. Ability to transfect cells by DNA incorporation in the NP.
		Ocular disease therapy	Ability to prolong drug residence times within the ocular mucus layer or retina. Alternative to the frequent application of high-drug concentration drops.
		Respiratory disease therapy	Mitigation of inflammatory responses in the respiratory tract.

## Data Availability

Not applicable.
